# Comprehensive two-dimensional gas chromatography-time of flight mass spectrometry as a tool for tracking roasting-induced changes in the volatilome of cold-pressed rapeseed oil

**DOI:** 10.1007/s00216-022-04486-6

**Published:** 2022-12-25

**Authors:** Natalia Drabińska, Aleksander Siger, Henryk Jeleń

**Affiliations:** 1 Food Volatilomics and Sensomics Group, Faculty of Food Science and Nutrition, Poznan University of Life Sciences, Poznań, Poland; 2 Department of Biochemistry and Food Analysis, Faculty of Food Science and Nutrition, Poznań University of Life Sciences, Poznań, Poland

**Keywords:** Roasting, Rapeseed oil, Cold-pressed, Volatilome, GC × GC-ToFMS

## Abstract

The aim of this study was to track changes in the volatilome of cold-pressed oil and press cakes obtained from roasted seeds and to combine it with the profile of non-volatile metabolites in a single study, in order to understand pathways of volatile organic compound (VOC) formation caused by thermal processing. Comprehensive two-dimensional gas chromatography-time of flight mass spectrometry was used for the analysis of VOCs in cold-pressed oils and corresponding press cakes obtained after roasting of seeds at 140 and 180 °C prior to pressing. Contents of primary metabolites (amino acids, saccharides, fatty acids) as well as selected secondary metabolites (glucosinolates, polyphenols) were determined, as many of them serve as precursors to volatile compounds formed especially in thermal reactions. After roasting, the formation of Maillard reaction products increased, which corresponded to the reduction of free amino acids and monosaccharides. Moreover, levels of the products of thermal oxidation of fatty acids, such as aldehydes and ketones, increased with the increasing temperature of roasting, although no significant changes were noted for fatty acids. Among sulphur-containing compounds, contents of the products and intermediates of methionine Strecker degradation increased significantly with the increasing temperature of roasting. Degradation of glucosinolates to nitriles occurred after thermal treatment. The results of this study confirmed that seed roasting before cold pressing has a significant effect on the volatiles, but also indicated roasting-induced changes in non-volatile metabolites of oil and press cake. Such an approach helps to understand metabolic changes occurring during rapeseed processing in cold-pressed oil production.

**Graphical Abstract:**

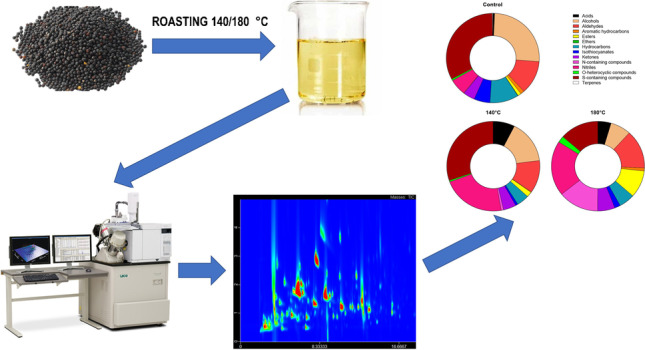

**Supplementary Information:**

The online version contains supplementary material available at 10.1007/s00216-022-04486-6.

## Introduction

Rapeseed oil is one of the most commonly consumed edible oils worldwide. The popularity of rapeseed as an oil crop is associated with its high oil yield and the beneficial profile of fatty acids with a substantial amount of n-3 acids (α-linolenic acid) [1]. The intake of unsaturated fatty acids has been repeatedly suggested to have a beneficial effect on human health [2, 3]. However, oils with a higher level of unsaturated fatty acids are more susceptible to oxidation. The oxidation of lipids is accelerated by the thermal treatment of oil, which can result in the formation of a wide range of aroma-active compounds affecting oil flavour [4]. Therefore, over the past few years, the interest in cold-pressed oils, which contain natural antioxidants having the ability to limit oxidation and consequently preserve the unique aroma and bioactive potential, is growing. The sensory properties of cold-pressed rapeseed oil are accepted by consumers; however, it has also been attempted to enrich its flavour even more.

One of the procedures affecting the aroma of cold-pressed oils is seed roasting before pressing [5]. This type of thermal treatment is usually performed to increase the oil yield by as much as 14% [6] and to increase even 120-fold the content of canolol, which is a potent antioxidant formed from sinapic acid by decarboxylation [5, 7–9]. Canolol is one of the few phenolic compounds which is lipid-soluble, which thus can improve the oxidative stability of oil and shelf-life of cold-pressed rapeseed oil. However, too long roasting at excessively high temperatures can result in an opposite effect with thermal degradation of canolol and decreased yield due to an insufficient moisture content of seeds [6, 9]. Roasting was found to affect also the content of other groups of compounds, such as tocopherols, sterols, and fatty acids [6, 8, 10].

All the changes in the metabolites of unprocessed rapeseed have an influence on the volatile compounds (VOCs) and consequently the sensory properties of cold-pressed oil. Roasting resulted in the formation of unique VOCs, with their type and contents being dependent on roasting conditions [11]. A typical result of thermal treatment is the formation of pleasant, roasty notes arising from the Maillard reaction. Kraljić et al. [6] found an increase in the presence of 2-methylpyrazine, 2,5-dimethylpyrazine, 2-ethylpyrazine, 2,6-dimethylpyrazine, 2-ethyl-6-methylpyrazine, 2-ethyl-3-methylpyrazine, 2-ethyl-5-methylpyrazine, and 2-ethyl-2,5-dimethylpyrazine as well as furfural, 5-methy-2-furfural, and 2-furanmethanol, which are typical representatives of Maillard reaction products. The increase in the Strecker aldehydes including 3-methylbutanal, 2-methylbutanal, and phenylacetaldehyde has also been reported after roasting of rapeseed seeds [6]. Dimethyl sulphide and dimethyl trisulfide, followed by 2,3-diethyl-5-methylpyrazine, 2,3-butanedione, octanal, 3-isopropyl-2-methoxypyrazine, phenylacetaldehyde, and 1-octene-3-one, were identified using gas chromatography–olfactometry as the key odorants of cold-pressed oil obtained from roasted seeds [5]. Oil from roasted seeds was found to be a richer source of VOCs compared to the control cold-pressed oil with 4-hydroxy-2,5-dimethylfuran-3(2H)-one (caramel-like), 2,3-diethyl-5-methylpyrazine (earthy), dimethyl trisulfide (cabbage-like), and 2-acetyl-1-pyrroline (popcorn-like) being the key odorants [12]. Consequently, the sensory analysis of cold-pressed oils obtained from roasted seeds characterised this oil as sweet, baked, nutty, roasted, and almond-like [5–7]. However, when roasting was performed too long or at excessively high temperatures, the unpleasant pungent, burnt, and coffee-like notes were perceived [13].

Although there are some studies on the aroma of cold-pressed oil from roasted seeds as well as the formation and/or degradation of non-volatile compounds in these oils, the formation of VOCs and their relations with non-volatiles are not fully elucidated. Therefore, this study aimed mainly to track changes in the volatilome of rapeseed oil related to seed roasting prior to oil pressing and refer these data to changes of non-volatile compounds, as many of them are precursors of detected volatiles. Also the same compounds were assessed in press cake to obtain a full picture of the distribution of these compounds between oil and press cake, thus providing more insight into the formation of VOCs by thermal processing.

## Materials and methods

### Materials

Seeds of rapeseed (*Brassica* *napus* L.) were purchased from Semco® (Śmiłowo, Poland). Before pressing, seeds were roasted for 10 min at 140 or 180 °C in the UFE55 Universal Oven with forced ventilation (Memmert KG, Schwabach, Germany). After roasting the water content was corrected to 8%. To obtain cold-pressed oils, seeds were pressed at room temperature using a Farmet Uno cold-pressing machine (Farmet, Czech Republic). The temperature inside the press was 60 °C and the temperature of the produced oil was 39 °C. The oil was centrifuged at 5000 rpm for 15 min and transferred directly to glass storage containers and stored at 4 °C in the dark. The obtained press cakes were defatted before the analyses.

### Non-volatile compounds

Tocochromanols, canolol, and polar phenolic compounds were analysed according to the method described by Siger et al. [14] using a Waters HPLC system (Waters, Milford, MA) consisting of a pump (Waters 600), a fluorimetric detector (Waters 474), and a photodiode array detector (Waters 2998 PDA). The XBridge TM C18 reversed-phase column (4.6 × 100 mm; 3.5 mm) (Waters, Milford, MA) was used for these analyses. The content of plastochromanol-8 (PC-8) was calculated following the method of Siger et al. [15].

Carotenoids were analysed according to the method provided by Wang et al. [16]. A high-performance liquid chromatography (HPLC) system equipped with the diode array detector (DAD) (UV–Vis, Waters, Milford, MA) was used.

Fatty acid composition in oils was determined as FAMEs (fatty acid methyl esters) using sodium methylate (CH_3_ONa, 0.4 N methanolic solution) for transesterification to methylated derivatives as described previously [5]. FAMEs were analysed using an Agilent Technologies 6890 GC gas chromatograph equipped with a flame ionisation detector (FID) and an autosampler.

The content of individual glucosinolates (GLS) was analysed according to the Official Journal of European Communities [17], as previously described in detail [18]. The separation of desulfo-GLS was performed using a 1100 series Agilent Technologies (HPLC) system with a DAD detector (Agilent Technologies, Waldbronn, Germany).

The content of individual amino acids was analysed using the method described by Drabińska [19]. Free amino acids (FAAs) were analysed using the EZ: Faast™ Kit for Free (Physiological) Amino Acids (Phenomenex, Aschaffenburg, Germany) according to the producer’s recommendations, using an Agilent 7890A/5975C gas chromatography/mass spectrometry (GC/MS) system equipped with a G45134 autosampler (Agilent Technologies, Santa Clara, CA, USA).

The extraction of sugars was performed as described previously [20]. Carbohydrates were determined using an Agilent Technologies 1100 series HPLC system with a refractive index detector (Agilent Technologies, Waldbronn, Germany).

### Volatile compounds

VOCs were analysed according to the method described previously [21] with a modification related to the second column. The extraction of VOCs was conducted using solid-phase microextraction (SPME) with 85-µm Carboxen™/polydimethylsiloxane (CAR/PDMS) (StableFlex) fibre purchased from Supelco (Bellefonte, PA, USA). One gram of oil or press cake was placed in 20-mL headspace vials, equilibrated for 6 min at 58 °C and then extracted for 38 min. VOCs were analysed by comprehensive two-dimensional gas chromatography-time of flight mass spectrometry (GC × GC-ToFMS) using an Agilent 6890 N gas chromatograph (Agilent Technologies, Palo Alto, CA, USA) equipped with a secondary oven and cryogenic (N2) modulator coupled to a PEGASUS 4D time-of-flight mass spectrometer (LECO, St. Joseph, MI). In this study, separation was performed on a SLB-5 (30 m × ⌀ 250 µm, d_f_0.25 µm; Supelco, Bellefonte, PA) as a first-dimension column and a SPB-50 (0.8 m × ⌀ 250 µm, d_f_ 0.25 µm; Supelco, Bellefonte, PA) as the second-dimension column. All the other parameters were the same as in the above-mentioned paper.

The ToFMS was operating in a mass range of *m*/*z* 33–383 and a detector voltage of 1700 V at 150 spectra/s. The data were collected and processed using a LECO ChromaTOF v.4.40 (LECO, St. Joseph, MI). The deconvoluted mass spectra of registered compounds were searched against the NIST library (version 2.0) of mass spectra. For the analyte match criteria, the following parameters were used: signal-to-noise (S/N) = 50 and minimum similarity spectral match ≥ 700. The peak areas of modulated peaks of the same unique mass were summarised for each annotated compound. Linear retention indices (LRI) were calculated relative to a series of alkanes (C8–C20) on the first-dimension column (DB-5 type). Based on the mass spectral library match and the comparison of LRI with that provided in the NIST database, the peaks were tentatively identified. Compounds that were present in fewer than 50% of samples were eliminated from statistical procedures.

### Statistical analysis

Data are expressed as means ± standard deviation (SD) of three or five replicates for non-volatile and volatile metabolites, respectively. The content of non-volatile compounds and the abundance of VOCs were compared by a one-way analysis of variance (ANOVA) test with Fisher’s LSD post hoc test using the STATISTICA version 13.3 (Statsoft, Tulsa, USA) software. Differences with a *p*-value lower than 0.05 were considered significant. Multivariate analysis of VOCs was performed using the SIMCA software package (version 16. Umetrics, Umea, Sweden). To identify differences between the groups, orthogonal partial least squares discriminant analysis (OPLS-DA) was used. To assess the contribution of each variable to the model and group separation variable importance in the projection (VIP) scores were measured.

## Results

### Volatilome of cold-pressed oils

**Fig. 1:**
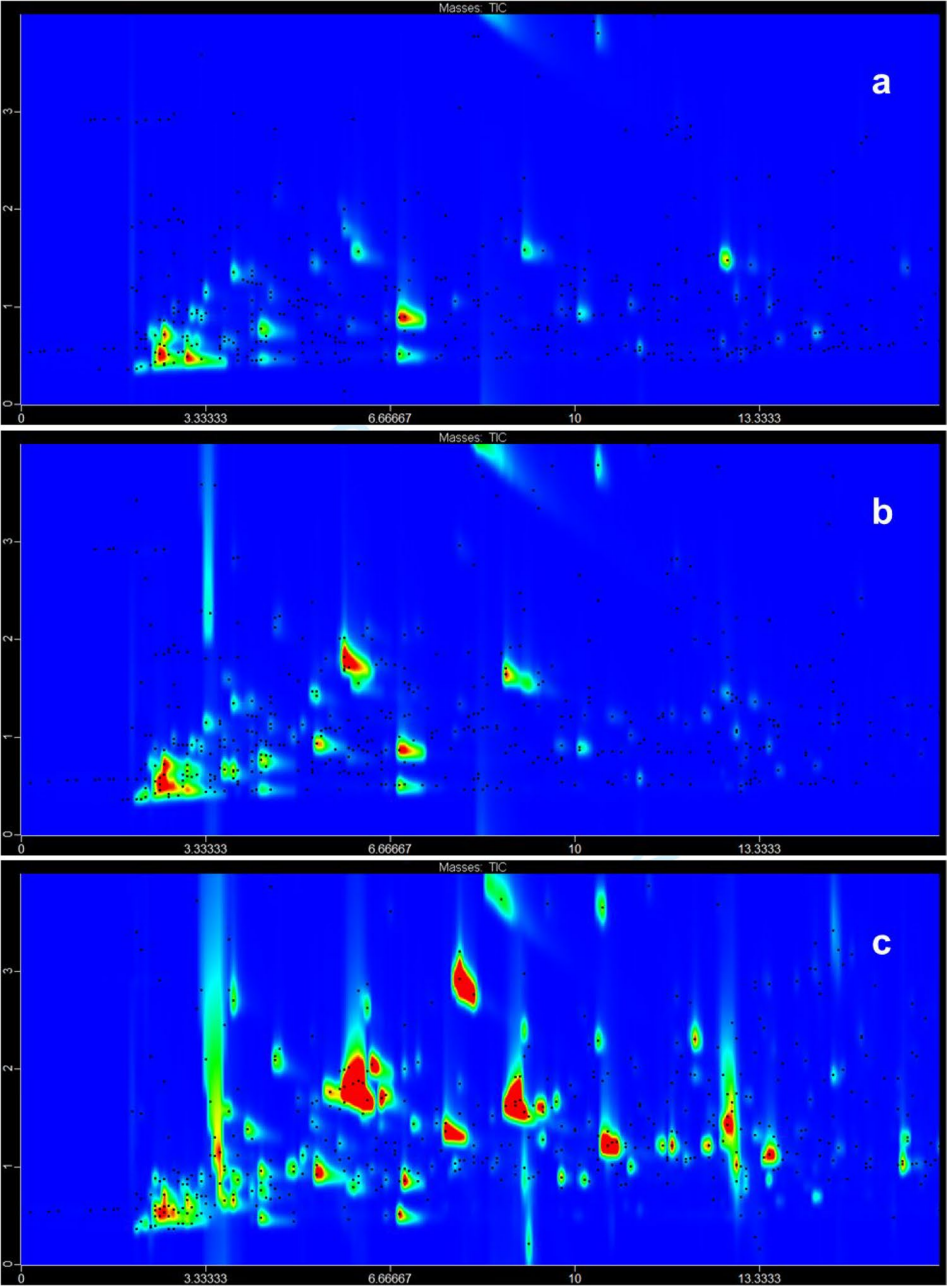
GC × GC-ToFMS chromatograms of volatile compounds of analysed cold-pressed oils. (**a**) Control oil, (**b**) oil obtained from seeds roasted at 140 °C, (**c**) oil obtained from seeds roasted at 180 °C

**Fig. 2:**
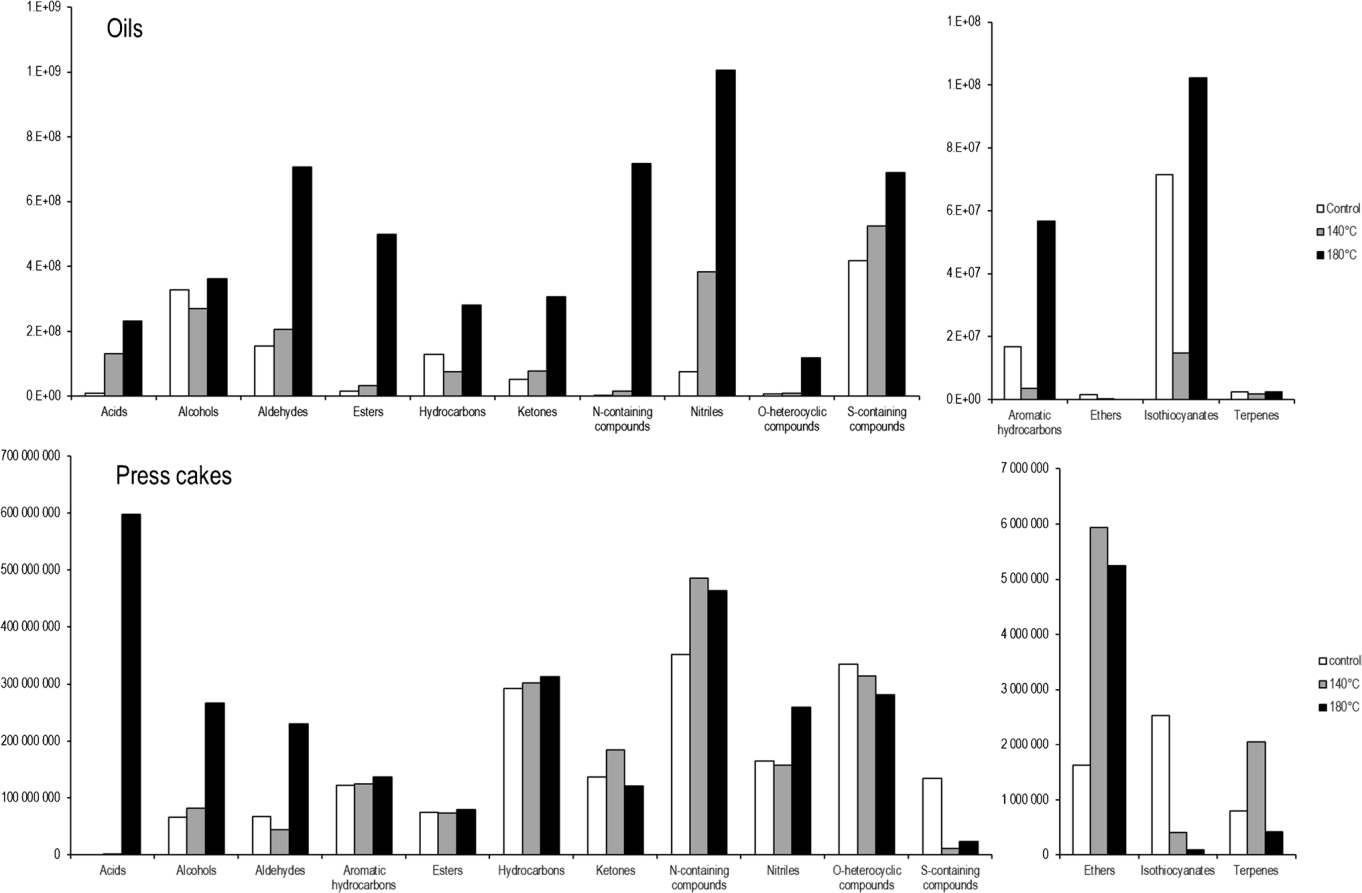
A comparison between the abundance of chemical classes in analysed oils and press cakes

In total, 436 VOCs were detected and tentatively identified in the analysed cold-pressed oils, including 10 acids, 54 alcohols, 38 aldehydes, 10 aromatic hydrocarbons, 28 esters, 3 ethers, 58 hydrocarbons, 7 isothiocyanates, 57 ketones, 47 N-containing compounds, 31 nitriles, 36 *O*-heterocyclic compounds, 43 S-containing compounds, and 14 terpenes (Supplementary Table 1). GC × GC chromatograms of VOCs in analysed oils are presented in Fig. 1. The distribution of the abundance between the chemical classes is presented in Fig. 2 and Supplementary Fig. 1. The most abundant chemical class in the control oil comprised S-containing compounds, followed by alcohols and aldehydes. In oil obtained from seeds roasted at 140 °C, the S-containing compounds were still dominant; however, the second dominant class were nitriles, followed by alcohols, aldehydes, and acids. In the oil roasted at 180 °C, nitriles were dominant compounds, followed by N-containing compounds, aldehydes, and S-containing compounds, and finally esters, acids, alcohols, hydrocarbons, and ketones. The abundance of acids, aldehydes, esters, ketones, N-containing compounds, nitriles, O-heterocyclic compounds, and S-containing compounds increased gradually with the increasing temperature of roasting. The abundance of alcohols, hydrocarbons, aromatic hydrocarbons, and isothiocyanates was greater in oils from seeds roasted at 180 °C, but lower after roasting at 140 °C compared to the control oil. Only the abundance of ethers decreased with the increasing temperature of roasting.

**Fig. 3:**
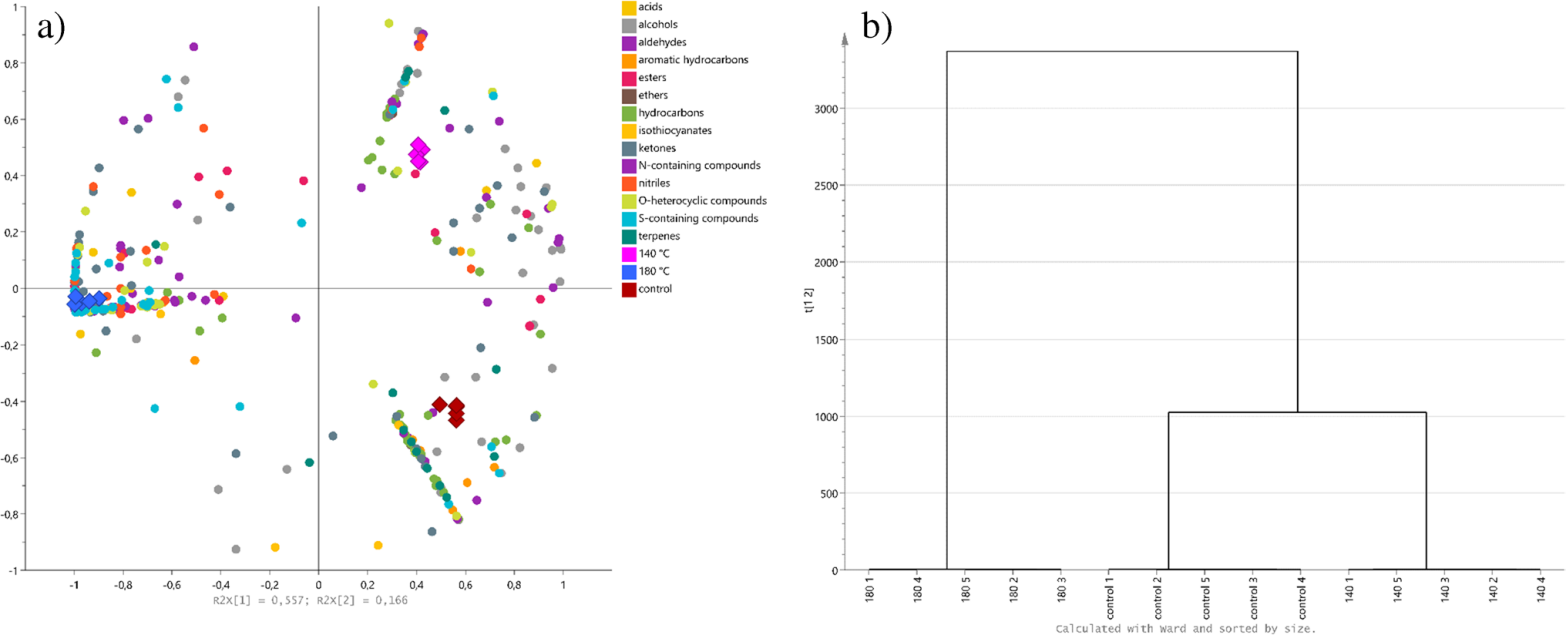
The multivariate analysis of VOCs detected in studied oils. **a**) Biplot from OPLS analysis; **b**) dendrogram from hierarchical analysis

The multivariate analysis showed a clear separation between the studied oils (Fig. 3a). The profile of the control oil and oil from seeds roasted at 140 °C was on the same side of the plot, while oil obtained from seeds roasted at 180 °C was positioned on the opposite side. It was confirmed by a hierarchical analysis, which revealed that oil from seeds roasted at 140 °C is more similar to the control than to the oil from seeds roasted at 180 °C (Fig. 3b). The compounds which contributed the most to the separation of oils (with the highest VIP score) were dihydro-5-methyl-2(3H)-furanone (NIST match: 931), 2-methyl-1-butanol (NIST match: 945), 1-hexanol (NIST match: 851), 1-(acetyloxy)-2-propanone (NIST match: 899), 2,5-dimethyl-1,5-hexadiene-3,4-diol (NIST match: 807), (3R)-( +)-3-acetamidopyrrolidine (NIST match: 879), 2-methyl-3-pentanone (NIST match: 863), 1-octen-3-ol (NIST match: 850 and confirmed with the standard), 4-methyl-1-pentanol (NIST match 810), and trimethyl-pyrazine (NIST match 853), all with VIP scores > 1.40. Considering that VIP > 1 is considered an important contributor, 233 VOCs met this criterion (4 acids, 40 alcohols, 20 aldehydes, 3 aromatic hydrocarbons, 7 esters, 1 ether, 16 hydrocarbons, 3 isothiocyanates, 34 ketones, 32 N-containing compounds, 18 nitriles, 19 *O*-heterocyclic compounds, 29 S-containing compounds, and 7 terpenes).

### Volatilome of press cakes

In total, 322 VOCs were detected and tentatively identified in analysed press cakes, including 4 acids, 41 alcohols, 22 aldehydes, 35 aromatic hydrocarbons, 24 esters, 5 ethers, 70 hydrocarbons, 2 isothiocyanates, 37 ketones, 20 N-containing compounds, 19 nitriles, 16 *O*-heterocyclic compounds, 15 S-containing compounds, and 12 terpenes (Supplementary Table 2). The distribution of the abundance between the chemical classes is presented in Fig. 2. In press cake obtained from the control oil, the dominant chemical classes were N-containing compounds, *O*-heterocyclic compounds, and hydrocarbons, followed by esters, nitriles, ketones, and aromatic hydrocarbons. A similar profile was determined for press cake from seeds roasted at 140 °C with dominant N-containing compounds, *O*-heterocyclic compounds, and hydrocarbons. The press cake originating from seeds roasted at 180 °C was characterised by the greatest abundance of acids, followed by N-containing compounds, alcohols, hydrocarbons, *O*-heterocyclic compounds, and nitriles. The abundance of acids, alcohols, aldehydes, and nitriles increased with the growing temperature of roasting. The opposite situation with a decrease in the abundance after roasting was detected for *O*-heterocyclic compounds, S-containing compounds, and isothiocyanates. The level of aromatic hydrocarbons, hydrocarbons, ketones, N-containing compounds, ethers, and terpenes was the highest in the press cake roasted at 140 °C. In contrast, the abundance of esters was similarly high in the press cakes obtained from the control and seeds roasted at 180 °C, while it was much lower in the press cake roasted at 140 °C.

**Fig. 4:**
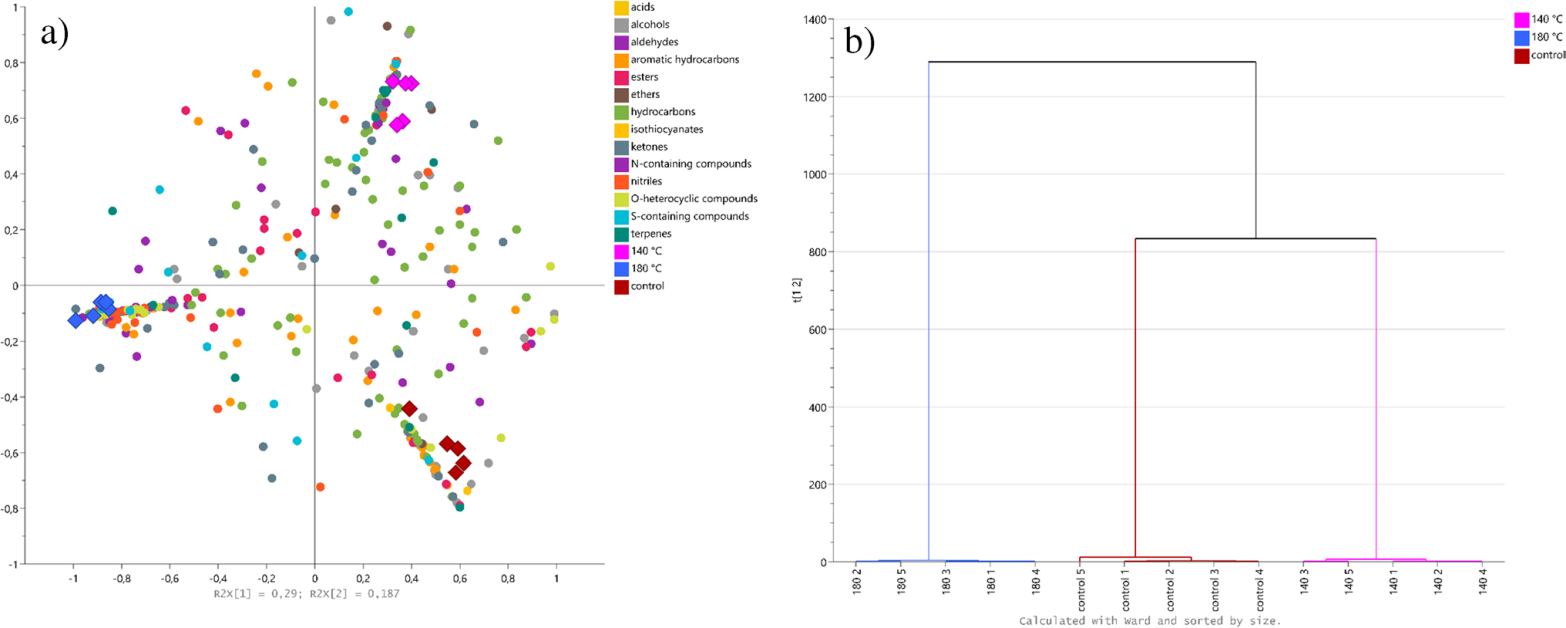
The multivariate analysis of VOCs detected in studied press cakes. **a**) Biplot from OPLS analysis; **b**) dendrogram from hierarchical analysis

The multivariate analysis of VOCs in press cakes (Fig. 4) showed similar trends to those observed in oils. Again, in the OPLS biplot, the control and press cakes from seeds roasted at 140 °C, although clearly separated, were placed on the one side, while press cakes from seeds roasted at 180 °C were positioned on the opposite side (Fig. 4a). The hierarchical analysis confirmed these results (Fig. 4b). The highest VIP score was determined for sulphur dioxide (NIST match: 959), 2,2-dimethyl-pent-4-yn-3-one (NIST match: 831), 2-methyl-2-propyl-oxirane (NIST match: 842), and *(E)-*2-hexen-1-ol (NIST match: 875) (all VIP > 1.40). A total of 154 VOCs had VIP scores > 1 and belonged to acids (3), alcohols (24), aldehydes (11), aromatic hydrocarbons (15), esters (9), ethers (3), hydrocarbons (19), isothiocyanates (1), ketones (20), N-containing compounds (11), nitriles (11), O-heterocyclic compounds (12), S-containing compounds (9), and terpenes (6).

### The profile of non-volatile metabolites

Contents of primary metabolites is presented in Table 1. Among the primary metabolites, there were 15 FAA and 6 saccharides detected and quantified in press cake and 8 fatty acids monitored in oils. Roasting of seeds before pressing resulted in a decrease in a majority of FAAs, irrespective of the applied temperature. For proline, asparagine, and glutamic acid, a downward trend was observed with the increase in roasting temperature. Tryptophan was found to be the most sensitive to high temperature, with losses of this compound by 22 and 49% after roasting at 140 and 180 °C, respectively.

**Table 1:** Contents of primary metabolites in press cakes and oil after roasting in comparison to the control

	Control	140 °C	180 °C
Press cake			
*Amino acids (µmol/g DM)*			
ALA	8.38 ± 0.42^a^	7.33 ± 1.07^a^	6.09 ± 2.11^a^
GLY	1.31 ± 0.32^a^	2.52 ± 0.81^a^	1.97 ± 0.58^a^
VAL	5.37 ± 0.24^a^	3.84 ± 0.08^b^	3.15 ± 1.05^b^
LEU	0.72 ± 0.07^a^	0.23 ± 0.02^b^	0.36 ± 0.20^b^
ILE	1.80 ± 0.68^a^	2.77 ± 0.28^a^	2.24 ± 0.74^a^
GABA	1.21 ± 0.12^b^	2.35 ± 0.04^a^	2.35 ± 0.42^a^
PRO	5.46 ± 0.79^a^	3.87 ± 0.56^a,b^	2.87 ± 0.75^b^
ASN	11.10 ± 1.52^a^	9.73 ± 0.77^a,b^	7.43 ± 1.10^b^
ASP	27.84 ± 2.59^a^	28.48 ± 2.15^a^	23.30 ± 3.56^b^
MET	0.51 ± 0.08^a,b^	0.60 ± 0.00^a^	0.47 ± 0.06^b^
GLU	38.35 ± 7.64^a^	26.32 ± 2.92^a,b^	22.69 ± 6.51^b^
PHE	2.29 ± 0.17^a^	2.17 ± 0.34^a^	2.07 ± 0.23^a^
GLN	3.01 ± 0.16^a^	3.64 ± 1.16^a^	1.66 ± 0.68^b^
TYR	0.82 ± 0.25^a^	0.64 ± 0.15^a^	0.63 ± 0.10^a^
TRP	0.98 ± 0.22^a^	0.76 ± 0.02^b^	0.50 ± 0.07^c^
⅀ amino acids	108.76 ± 13.26^a^	95.25 ± 2.35^a,b^	77.78 ± 16.48^b^
*Saccharides (mg/g DM)*			
Glucose	10.95 ± 2.77^b^	12.54 ± 1.78^a^	6.09 ± 0.44^c^
Galactose	3.18 ± 0.95^b^	4.07 ± 0.82^a^	0.59 ± 0.13^c^
Fructose	5.23 ± 1.43^a,b^	5.54 ± 0.64^a^	3.88 ± 0.35^b^
Sucrose	90.23 ± 27.27^b^	123.16 ± 18.95^a^	114.75 ± 8.16^a^
Raffinose	7.23 ± 2.32^b^	10.27 ± 1.84^a^	11.52 ± 1.47^a^
Stachyose	52.43 ± 6.71^b^	83.98 ± 12.54^a^	90.39 ± 6.06^a^
⅀ sugars	169.26 ± 41.45^b^	239.56 ± 36.57^a^	227.21 ± 16.60^a^
Oil			
FAME** (mg/g of oil)			
C16:0	15.82 ± 0.43^a^	14.38 ± 0.95^a^	14.79 ± 0.97^a^
C16:1	0.72 ± 0.14^a^	0.69 ± 0.04^a^	0.74 ± 0.04^a^
C18:0	5.82 ± 0.27^a^	5.33 ± 0.37^a^	5.56 ± 0.38^a^
C18:1	244.97 ± 12.76^a^	220.46 ± 15.17^a^	230.84 ± 15.83^a^
C18:2	67.16 ± 3.61^a^	60.32 ± 4.13^a^	62.42 ± 4.29^a^
C18:3	25.85 ± 1.40^a^	23.24 ± 1.62^a^	24.34 ± 1.68^a^
C20:0	1.44 ± 0.10^a^	1.57 ± 0.09^a^	1.65 ± 0.11^a^
C20:1	3.37 ± 0.10^a^	3.56 ± 0.29^a^	3.79 ± 0.27^a^
⅀ FAME	365.13 ± 18.53^a^	329.55 ± 22.66^a^	344.13 ± 23.56^a^

^*^Different letters in the same row indicate a significant difference (*p* < 0.05) (LSD Fisher, ANOVA)

^**^Fatty acid methyl esters

The content of sugars was found to be dependent on the applied temperature. The content of individual monosaccharides was the highest after roasting at 140 °C and the lowest after roasting at 180 °C. For the other saccharides, which are composed of 2, 3, and 4 sugar units, the difference between roasting at 140 and 180 °C vanished. The tendency to increase the content of sugars with an increasing number of saccharide units at higher temperatures, although not statistically significant, was observed.

Fatty acids expressed as fatty acid methyl esters (FAMEs) were not affected by roasting. Although noticed lower contents of FAMEs in roasted oils was noted compared to the control oil, the differences were not statistically significant.

Contents of secondary hydrophilic metabolites in press cakes and oils are presented in Table 2. Due to the hydrophilic nature of these compounds, a majority of them were detected only in press cakes. Only 4 polyphenols were detected in oils. Notably, the content of polyphenols in oils increased with the growing temperature of roasting and in the oil obtained from seeds roasted at 180 °C, 2 new polyphenols, not detected in the control oil, were found. More than a twofold higher content of polyphenols was observed for oil roasted at 180 compared to 140 °C.

**Table 2:** Contents of hydrophilic secondary metabolites in oils and press cakes after roasting in comparison to the control

	Press cake			Oil		
	Control	140 °C	180 °C	Control	140 °C	180 °C
*Glucosinolates (nmol/g DM)*						
Glucoiberin	57.25 ± 2.03^b^	71.99 ± 0.07^a^	49.46 ± 0.05^b^	/	/	/
Progoitrin	9458.77 ± 386.16^b^	11,580.03 ± 11.58^a^	5807.63 ± 5.81^c^	/	/	/
Glucoraphanin	181.33 ± 8.70^b^	215.49 ± 0.22^a^	104.61 ± 0.10^c^	/	/	/
Sinigrin	126.61 ± 3.92^a^	126.11 ± 0.13^a^	85.22 ± 0.09^b^	/	/	/
Gluconapin	3378.28 ± 126.06^b^	4655.14 ± 4.66^a^	2572.9 ± 2.57^c^	/	/	/
Glucobrassicanapin	1387.44 ± 56.18^b^	1692.52 ± 1.69^a^	906.23 ± 0.91^c^	/	/	/
Glucotropeaolin	55.18 ± 3.31^b^	64.65 ± 0.06^a^	27.45 ± 0.03^c^	/	/	/
Glucoerucin	127.74 ± 6.06^b^	154.51 ± 0.15^a^	82.1 ± 0.08^c^	/	/	/
Glucobrassicin	880.81 ± 18.05^b^	985.96 ± 0.99^a^	397.71 ± 0.40^c^	/	/	/
Neoglucobrassicin	589.4 ± 41.54^a^	624.52 ± 0.62^a^	171.47 ± 0.17^b^	/	/	/
⅀ glucosinolates	16,242.81 ± 635.45^b^	20,170.92 ± 20.17^a^	10,204.78 ± 10.20^c^	/	/	/
*Polyphenols (µg/g DM)*						
Caffeic acid	0.51 ± 0.03^b^	1.02 ± 0.10^a^	0.35 ± 0.05^b^	/	0.47 ± 0.01^B^	2.33 ± 0.03^A^
Sinapin	183.71 ± 2.28^a^	182.52 ± 1.05^a^	160.86 ± 0.15^b^	1.10 ± 0.02^C^	1.60 ± 0.02^B^	2.68 ± 0.04^A^
Sinapoyl-glucose	39.48 ± 0.50^b^	46.20 ± 0.52^a^	8.94 ± 0.12^c^	/	/	/
Ferulic acid	0.70 ± 0.08^b^	0.93 ± 0.02^a,b^	1.07 ± 0.12^a^	/	/	/
3-Dihexoside-7-sinapoyl-hexoside kaempferol	18.79 ± 0.22^b^	23.06 ± 1.12^a^	11.03 ± 0.11^c^	/	/	/
*trans*-Sinapic acid	10.01 ± 0.03^b^	12.85 ± 0.30^a^	6.64 ± 0.21^c^	0.14 ± 0.01^B^	0.11 ± 0.01^B^	0.35 ± 0.03^A^
*cis*-Sinapic acid	9.24 ± 0.34^b^	15.37 ± 0.37^a^	3.83 ± 0.18^c^	/	/	/
3-O-Hexoside kaempferol	4.48 ± 0.08^b^	6.63 ± 0.11^a^	2.81 ± 0.04^c^	/	/	/
Sinapoyl malate	6.10 ± 0.14^b^	8.65 ± 0.12^a^	4.00 ± 0.06^c^	/	/	/
3-Hexoside-7-sinapoyl-hexoside kaempferol	3.60 ± 0.07^b^	5.37 ± 0.35^a^	2.16 ± 0.09^c^	/	/	/
Sinapic acid methyl ester	14.17 ± 0.06^b^	19.80 ± 0.38^a^	5.55 ± 0.10^c^	/	/	0.18 ± 0.01
1,2-Disinapoyl-dihexoside	24.49 ± 0.24^b^	29.87 ± 0.35^a^	9.13 ± 0.04^c^	/	/	/
1,2-Disinapoyl-hexoside	7.10 ± 0.27^b^	9.44 ± 0.18^a^	2.15 ± 0.10^c^	/	/	/
1,2,2′-Trisinapoyl-dihexoside	3.70 ± 0.09^b^	5.28 ± 0.21^a^	1.38 ± 0.14^c^	/	/	/
⅀ polyphenols	326.06 ± 1.77^b^	366.96 ± 0.50^a^	219.87 ± 0.77^c^	1.24 ± 0.04^C^	2.18 ± 0.03^B^	5.54 ± 0.04^A^

^*^Different letters (small for press cake and capital for oil) in the same row indicate a significant difference (*p* < 0.05) (LSD Fisher, ANOVA)

In press cakes, the presence of 10 GLS and 14 polyphenols was detected. The total contents of both these secondary metabolites was the highest in the press cake from seeds roasted at 140 °C. The roasting at a higher temperature resulted in a degradation of all GLS and a majority of polyphenols. Only the contents of ferulic acid increased with the growing temperature of roasting.

Contents of lipophilic secondary metabolites in studied oils and press cakes are presented in Table 3. In total, 10 lipophilic compounds, including 4 carotenoids, 4 tocopherols, plastochromanol-8, and canolol, were detected in both press cakes and oils. In press cakes, the contents of carotenoids decreased with the increasing temperature of treatment. Only the contents of 13-*Z*-β-carotene was the highest in press cake from seeds roasted at 180 °C. However, the contents of this compound was generally small; therefore, it did not affect the total contents of carotenoids. Tocopherols were not markedly affected by roasting and the overall contents of tocopherols did not differ between the studied press cakes. Only the contents of β- and γ-tocopherol increased with a rise in roasting temperature. The contents of plastochromanol-8 decreased with the increasing temperature of roasting. The most significant changes were observed for canolol, which contents increased 6- and 17-fold after seed roasting at 140 and 180 °C, respectively.

**Table 3:** Contents of lipophilic secondary metabolites in oils and press cakes after roasting in comparison to the control

	Press cake			Oil		
	Control	140 °C	180 °C	Control	140 °C	180 °C
*Carotenoids (µg/g DM)*						
Lutein	8.82 ± 0.01^a^*	6.41 ± 0.12^b^	4.90 ± 0.08^c^	6.85 ± 0.01^A^	4.19 ± 0.03^B^	3.37 ± 0.02^C^
Zeaxanthin	0.18 ± 0.00^a^	0.18 ± 0.01^a^	0.12 ± 0.00^b^	0.38 ± 0.01	/	/
β-Carotene	1.06 ± 0.01^a^	0.87 ± 0.01^a^	0.43 ± 0.22^b^	1.14 ± 0.02^B^	1.27 ± 0.01^A^	1.07 ± 0.01^C^
13-*Z*-β-Carotene	0.27 ± 0.01^b^	0.29 ± 0.00^a,b^	0.31 ± 0.01^a^	0.27 ± 0.00^C^	0.42 ± 0.00^B^	0.63 ± 0.01^A^
⅀ carotenoids	10.34 ± 0.00^a^	7.75 ± 0.14^b^	5.76 ± 0.15^c^	8.64 ± 0.02^A^	5.88 ± 0.02^B^	5.08 ± 0.03^C^
*Tocopherols (µg/g DM)*						
α-Tocopherol	1.44 ± 0.04^a,b^	1.39 ± 0.01^b^	1.47 ± 0.01^a^	3.54 ± 0.02^A^	3.46 ± 0.00^B^	3.36 ± 0.01^C^
β-Tocopherol	0.01 ± 0.00^b^	0.02 ± 0.00^a^	0.01 ± 0.00^a^	0.01 ± 0.00^A^	0.01 ± 0.00^A^	0.01 ± 0.00^A^
γ-Tocopherol	0.87 ± 0.01^c^	0.93 ± 0.01^b^	0.98 ± 0.01^a^	3.48 ± 0.02^A^	3.11 ± 0.02^B^	3.15 ± 0.02^B^
δ-Tocopherol	0.02 ± 0.00^a^	0.02 ± 0.00^a^	0.02 ± 0.00^a^	0.07 ± 0.00^A^	0.05 ± 0.00^B^	0.05 ± 0.00^B^
⅀ tocopherols	2.69 ± 0.04^a^	2.68 ± 0.01^a^	2.76 ± 0.03^a^	7.35 ± 0.02^A^	6.90 ± 0.03^B^	6.95 ± 0.01^B^
*Other compounds (µg/g DM)*						
Plastochromanol-8	0.35 ± 0.01^a^	0.32 ± 0.01^a,b^	0.28 ± 0.02^b^	0.25 ± 0.02^B^	0.26 ± 0.00^B^	0.38 ± 0.02^A^
Canolol	0.37 ± 0.01^c^	2.25 ± 0.02^b^	6.37 ± 0.07^a^	0.04 ± 0.00^C^	0.93 ± 0.02^B^	4.98 ± 0.05^A^

^*^Different letters (small for press cake and capital for oil) in the same row indicate a significant difference (*p* < 0.05) (LSD Fisher, ANOVA)

For oil, similar tendencies to those in press cakes were also observed. The content of carotenoids decreased after thermal treatment of seeds before pressing, with zeaxanthin not detected in any of the roasted oils. Only the contents of 13-*Z*-β-carotene, similarly to press cake, increased with the rise in roasting temperature. More marked changes were observed for tocopherols in oils compared to press cake. After roasting, the overall contents of tocopherols decreased, irrespective of the processing temperature. The greatest changes were observed for α-tocopherol, which level gradually decreased with the increasing temperature of roasting. The contents of plastochromanol-8, in contrast to the press cake, increased after roasting at the highest analysed temperature. And finally, the canolol level increased, similarly to press cake, with the increasing temperature of roasting and its content increased 23- and 125-fold after roasting at 140 and 180 °C, respectively, compared to the control, unprocessed oil.

## Discussion

The strategy of a simultaneous analysis of the volatilome and the metabolome opens a new perspective for correlating the volatile and non-volatile metabolites to provide insight into the metabolic pathways occurring during food processing. The results of this study confirmed that seed roasting before cold pressing has a tremendous effect on both the volatilome and non-volatile metabolome of oils and press cakes. For many of the analysed VOCs, an association may be observed between them and contents of the potential precursors. To the best of our knowledge, this study presents the most comprehensive profile of VOCs in cold-pressed rapeseed oil from roasted seeds with > 400 VOCs detected and tentatively identified in a single study.

The most typical change caused by roasting is the intensification of the Maillard reaction resulting in the formation of pyrazines, furanones, and pyrroles [22, 23]. The precursors of the Maillard reaction are amino acids and reducing sugars. Both these groups were significantly reduced after roasting at the highest temperature. Moreover, formation of aldehydes being derivatives of 1H-pyrrole-2-carboxaldehyde as well as 5-methyl-2-furancarbohyaldehyde, 2-furaldehyde, and furfural was observed in oil from seeds roasted at the high temperature, which can also be explained by the Maillard reaction. Contents of 2-methyl-2-butanal, 3-methyl-2-butanal, and 2-methyl-propanal, which are products of the Strecker degradation of amino acids induced by heat, increased significantly with the increasing temperature of roasting. It is in agreement with a previous study, in which these aldehydes were dominant after roasting of rapeseed seeds [5]. Additionally, levels of other aldehydes including (*E,E*)-2,4-heptadienal, 2-butenal, ethyl-2-butenal, 2-methyl-2-butenal, 2-hexenal, 2-octenal, acetaldehyde, benzaldehyde, benzenacetaldehyde, hexanal, and nonanal increased after roasting. Many of these compounds are formed during the oxidation of lipids, especially the aldehydes with double bonds (mostly 2-alkenals and 2,4-alkadienals), which are derived from less stable, unsaturated fatty acids [24]. In the press cake, fewer aldehydes were detected compared to oil; however, their abundance also increased after roasting and corresponded to the Maillard reaction and lipid oxidation. Although in this study the content of FAMEs did not decrease significantly, a clear downward trend tendency can be noted, which can explain the formation of many of the aldehydes. Furfural was the dominant aldehyde in the press cake from seeds roasted at 180 °C, and compared to the control press cake, its level increased 42-fold, indicating intensification of the Maillard reaction. The most prominent pathway for thermal formation of furfural in the Maillard reaction is the dehydration of 3-deoxyosone, which especially at pH < 5 is formed from Amadori compounds. Interestingly, there was a considerable difference in the abundance of aldehydes between the press cakes from seed roasted at 140 and 180 °C, which can be explained by the temperature dependence of the Maillard reaction. High temperature speeds up the Maillard reaction due to an increase in the rate of chemical reactions as well as acceleration of water evaporation, which increase the concentration of substrates in the matrix. Moreover, pH of the medium decreases with increasing temperature after roasting [25], which is considered to facilitate the formation of flavour compounds such as furfural [26].

A different observation was noted for ketones, which abundance increased in oils with the increased temperature of roasting, while in press cake it decreased after seed roasting at 180 °C. In oil, the biggest roasting-induced increase was observed for diketones, especially vicinal diketones, being also Maillard reaction intermediates formed in the fission of reductones (2,3-pentanedione as well as 2,4-pentanedione, 2,5-hexanedione), unsaturated ketones (3-octene-2-one, 3-penten-2-one, 4-methyl-3-penten-2-one, 6-methyl-5-hepten-2-one), hydroxyketones (3-hydroxy-2-butanone, 2-hydroxy-3-pentanone, 1-hydroxy-2-propanone), and ketones with the furanyl group (1-(2-furanyl)-1-propanone, 1-(2-furanyl)-ethanone). Compounds with the furanyl group as well as diketones are typical products of the Maillard reaction detected after roasting [27]. At the same time, the level of aliphatic C6-C7 ketones (2-heptanone, 2-hexanone) decreased after roasting, which can be explained by the reduction to corresponding alcohols. For instance, the abundance of 2-hexanol increased after roasting around 60-fold, which can be associated with the reduction of 2-hexanone.

Alcohols can be formed via the Ehrlich pathway from amino acids or the oxidation of lipids [28]. In this study, roasting did not have a substantial effect on the overall abundance of alcohols in oil. However, a significant roasting-induced increase was noted for 2-hexanol, 2-furanmethanol, and alcohols with double bonds. Although in this study no significant reduction in FAMEs was observed, a decreasing trend was noted, which could be enough for the formation of volatile derivatives. Thermal degradation of saturated fatty acids results in the formation of hydrocarbons, which increased in this study in the oil from seeds roasted at 180 °C, especially 2-methyl-1,5-heptadiene, heptane, octane, and 3,3-dimethyl-1-butyne, which is in agreement with a previous model study [29].

An interesting observation was the high abundance of acids, mainly acetic acid, after roasting at a high temperature. A similar observation was previously reported for roasted coffee [30]. The main precursor of acetic acid is sucrose, hydrolysed to glucose and fructose, which directly contribute to the aliphatic acid formation. Monosaccharides rearrange with the 1,2-endiol as an intermediate in the Lobry-de-Bruyn-van-Eckenstein reaction, which then leads to the formation of acetic acid [30]. In this study, the content of sucrose increased after roasting; thus, this compound could not contribute to the increase in acetic acid content. However, contents of its hydrolysis products, glucose and fructose, which are directly metabolised to acetic acid, decreased with the increasing temperature of roasting; thus, it can be assumed that their transformation could result in the formation of the aforementioned acid.

Many esters were formed after roasting at the highest temperature. Esters can be formed in various pathways, originating from the oxidation of fatty acids and degradation of amino acids [28]. It is considered that branched fatty acid ethyl esters are related to nitrogen metabolism, while straight-chain analogues are derived from lipid metabolism [31]. In coffee roasting, the profile of aroma compounds, with a great number of esters, is formed via epimerisation, dehydration, and transesterifications (acyl migration being a special case) [32]. A similar phenomenon can be expected in roasted oils. Among the detected esters, lactones can be formed from phenolic acids, carbohydrates, and carotenoids [27, 33]. Two parallel reactions such as the enolate Spengler-Pfannenstiel degradation and the Cannizzaro reaction of α-dicarbonyl compounds can lead to various saccharic acids, which may form lactones [33]. In this study, the reduction in both polyphenolic compounds and carotenoids was observed at the highest roasting temperature, which can be a possible source of lactones. Carotenoids could also be degraded to terpenes [34] and methylglyoxal [35], the latter being detected only after roasting at 180 °C. Notably, after roasting, an increase in 13-*Z*-β-carotene was noted, which can be explained by heat-induced isomerisation, previously reported for virgin olive oil [36]. In another model study, one of the carotenoids not detected in this study, namely lycopene, was degraded with the formation of terpenes, but also alcohols (1-pentanol), aldehydes (hexanal, (*E*)-2-octen-1-al), and ketones (4-hydroxy-4-methyl-2-pentanone, 6-methyl-5-hepten-2-one) [37]. Hence, considering the comparable structure of carotenoids, similar degradation pathways could be expected in our study.

A reduction of polyphenolic compounds after heat treatment can lead to the formation of esters as aforementioned, but also volatile phenolic compounds [27]. In our study, the presence of 4-methoxy-4-vinylphenol and phenol was noted after roasting at 180 °C, which can be explained by the degradation of polyphenols. Moreover, an important product of thermal decarboxylation of sinapic acid is canolol, which level increased significantly in oils from roasted seeds, as repeatedly reported previously [6, 38].

Among sulphur-containing compounds, levels of the products and intermediates of methionine Strecker degradation, including dimethyl trisulfide, dimethyl disulphide, methanethiol, and 3-(methylthio)-propanal, increased significantly with the rise in roasting temperature. In the oil obtained from seeds roasted at 180 °C, the presence of various thiazoles and thiophenes was recorded. These compounds can also be formed as products of the Maillard reaction [27].

In general, in the cold-pressed rapeseed oil, 4-isothiocyanate-1-butene, which is the breakdown product of gluconapin, is the dominant GLS derivative. Both nitriles and isothiocyanates were the most abundant in oil from seeds roasted at 180 °C, which can be associated with the highest degradation of GLS after roasting at this temperature. This could be explained by the degradation of GLS, which was observed after processing at 180 °C. However, in the oil obtained from the roasted seeds, the formation of nitrile compounds dominated, due to the inactivation of enzymes and thermal degradation of GLS. Previous studies showed that high temperature leads to the shifting of the GLS degradation from isothiocyanates to nitriles [39, 40]. In a study of Jing et al. [11], roasting of seeds for 40 min at 150 °C increased the content of nitriles, including 2-butenenitrile, 2-methyl-butanenitrile, 5-(methylthio)-pentanenitrile, acetonitrile, and octanenitrile, from 0.26 to 1.56 mg/kg to 99.14–267.97 mg/kg, and the nitrile compounds were considered as key odourants (5-(methylthio)-pentanenitrile, odour activity value (OAV) > 9.2; octanenitrile, OAV > 5.8; 2-butenenitrile, OAV > 2.5). Mao et al. [13] reported that thermal treatment leads to the formation of mainly low-carbon nitriles, which significantly affect the overall aroma of oil. Interestingly, roasting at 140 °C, which is still a high temperature, did not degrade GLS to that extent, and in these samples, the greatest abundance of GLS was observed. As reported by Kraljić et al. [6], the formation of nitriles is highly dependent on the temperature of processing, and in this study, the content of 2-methyl-2-butenenitrile, 2-methyl-5-hexanenitrile, and heptanenitrile increased with the rise in the temperature of treatment.

In summary, this study showed a substantial influence of seed roasting process on the profile of volatile compounds detected in cold-pressed rapeseed oil. GCxGC-ToFMS with an unrivalled peak capacity allows for spatial separation and identification of deconvoluted peaks of hundreds of volatile compounds, providing data for untargeted analysis and technological treatment comparison. Moreover, the results of this study showed that combined volatilomics and non-volatile metabolomics, where among other compounds the main precursors of volatiles are monitored, can help to understand the formation of VOCs in food matrices. The chemometric analysis showed that the temperature has a great impact on the profile of VOCs and the 40 °C difference tremendously changes the occurring metabolic pathways. After roasting at the highest temperature (180 °C), the very intensive Maillard reaction occurred resulting in the formation of various new compounds such as pyrazines, furan-derivatives, thiazoles, and diketones. Additionally, the products of lipid oxidation were formed after seed roasting prior to pressing. Among sulphur-containing compounds, levels of the products and intermediates of methionine Strecker degradation increased significantly with the rise in roasting temperature. Moreover, the degradation of GLS favoured nitrile formation after the thermal processing of seeds. The results of this study confirmed that seed roasting before cold pressing has a considerable effect on the volatilome and non-volatile metabolome of oils and press cakes; thus, an explanation of the metabolic pathways involved in their formation/degradation was proposed.

## Supplementary Information

Below is the link to the electronic supplementary material.

Supplementary file1 (PDF 974 KB)
