# Supplementary material for: Comprehensive two-dimensional gas chromatography-time of flight mass spectrometry as a tool for tracking roasting-induced changes in the volatilome of cold-pressed rapeseed oil
Source: Anal Bioanal Chem. 2022 Dec 25;415(13):2523–34. doi: 10.1007/s00216-022-04486-6 (PMC10149463; doi:10.1007/s00216-022-04486-6)
Supplement: Supplementary file 1 — Supplementary file1 (PDF 974 KB) [file 216_2022_4486_MOESM1_ESM.pdf]

Comprehensive two-dimensional gas chromatography–time of flight mass spectrometry as a tool for tracking roasting-induced changes in the volatilome of cold-pressed rapeseed oil

Natalia Drabińska[1], Aleksander Siger[2], Henryk Jeleń[1]

Supplementary Figure 1. Pie charts summarizing the percentage contribution of the chemical classes into the overall volatilome in analysed oils.

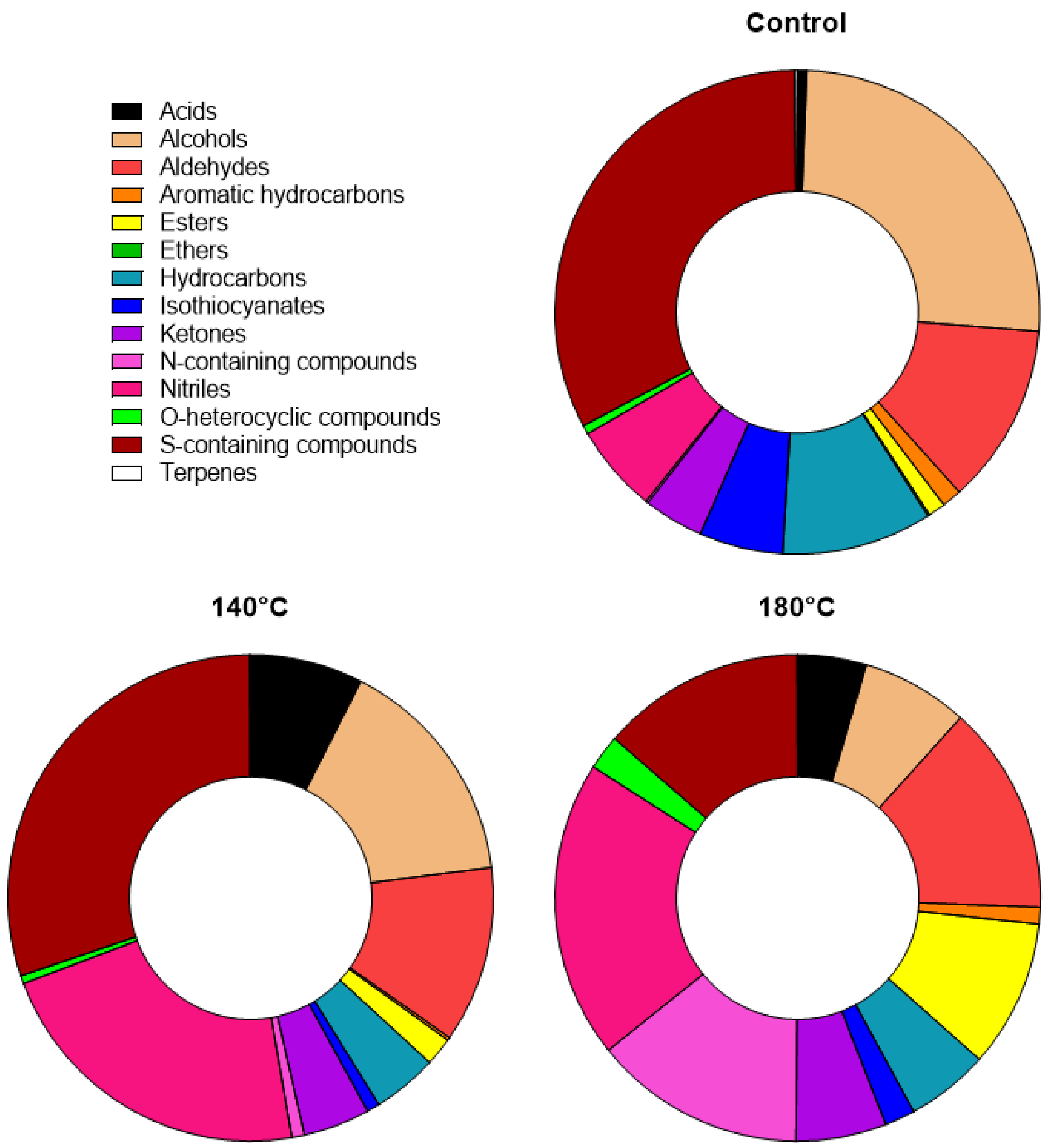

Supplementary Table 1. Volatile compounds tentatively identified by GC×GC-ToFMS in cold-pressed oils obtained from roasted seeds. Variable Importance in Projection (VIP) scores for each compound inform about the contribution to the separation between the studied oils. Results expressed and mean of peak areas of 5 replicates ± SD.

	RT[1] (min)	LRI[2]	Control	140 °C	180 °C	VIP
Acids						
4-Methyl-3-pentenoic acid	12.906 , 2.198	996	43,705 ± 4,873[b]	69,867 ± 2,041[a]	ND	1.16
Acetic acid*	3.330 , 2.178	617	5,428,746 ± 1,557,386[c]	120,846,616 ± 38,030,509[b]	198,679,127 ± 17,892,710[a]	0.96
(Acetyloxy)-acetic acid	6.0787 , 2.732	788	ND	4,188,041 ± 14,007	ND	1.01
Acetic acid, hydrazide	3.164 , 0.521	685	ND	ND	3,325,839 ± 104,039	0.42
Acetic anhydride	6.661 , 3.571	807	ND	4659224 ± 731957[b]	8,071,697 ± 170,127[a]	1.00
1-Amino-cyclopropanecarboxylic acid	3.663 , 1.003	713	ND	ND	961,579 ± 224,041	0.74
Methanesulfonic anhydride	2.747 , 1.861	<500	426,833 ± 122,229[a]	730,941 ± 12,001[b]	ND	0.90
Methyl propionate*	3.330 , 0.627	703	ND	ND	1,789,513 ± 109,793	0.74
Propanoic acid*	4.912 , 4.198	753	ND	ND	3,453,036 ± 1,000,168	0.75
Propanoic acid, anhydride	4.163 , 1.287	729	2,230,632 ± 581,272[b]	333,300 ± 362,237[c]	15,393,949 ± 1,423,448[a]	1.07
Alcohols						
(*R*)-(-)-2-Pentanol*	4.496 , 1.148	739	14,254,924 ± 478,835	ND	ND	1.36
1,3-Propanediol	9.326 , 3.359	889	1,138,911 ± 283,895[a]	1,214,712 ± 81,397[a]	ND	1.04
1,5-Hexadien-3-ol	12.990 , 0.898	998	ND	ND	221,175,117 ± 42,568,184	0.74
2,5-Dimethyl-1,5-hexadiene-3,4-diol	13.989 , 1.736	1029	ND	98,738 ± 6,367	ND	1.41
3,7-Dimethyl-1,6-octadien-3-ol*	16.570 , 1.234	1109	43,041 ± 14,683[a]	28,726 ± 413[a]	ND	0.83
1-Butanol*	3.830 , 1.353	666	29,577,546 ± 819,553[a]	31,394,065 ± 873,166[a]	1,731,839 ± 228,796[b]	1.08
2-Methyl-1-butanol*	5.329 , 1.426	745	ND	20,470,287 ± 394,788[a]	638,862 ± 197,718[b]	1.42
3-Methyl-1-butanol*	5.246 , 1.452	749	8,390,371 ± 247,545	ND	ND	1.36
1-Heptanol*	12.490 , 1.439	983	831,752 ± 28,360[a]	905,493 ± 25,215[a]	ND	0.78
1-Hexanol*	9.159 , 1.518	883	54,263,651 ± 2,666,596[a]	732,870 ± 175,312[b]	48,942,888 ± 7,452,094[a]	1.42
2-Ethyl-1-hexanol*	14.405 , 1.241	1042	376,544 ± 42,901[b]	349,411 ± 72,012[b]	509,838 ± 7,2453[a]	0.84
1-Methylcycloheptanol	16.987 , 1.412	1123	ND	60,689 ± 7,377	ND	1.22
1-Octanol*	15.738 , 1.366	1083	198,274 ± 49,376[a]	269,357 ± 127,306[a]	ND	0.96
2-Butyl-1-octanol	14.905 , 0.561	1057	65,431 ± 12,495[a]	37,513 ± 11,417[a]	ND	1.00
1-Octen-3-ol*	12.823 , 1.379	993	ND	4,801,194 ± 254,362	ND	1.41

1-Pentanol*	6.078 , 1.558	788	50,397,135 ± 1,364,565[a]	22,472,038 ± 479,540[b]	6,902,294 ± 329,098[c]	1.22
4-Methyl-1-pentanol	9.159 , 1.544	883	ND	57,872,725 ± 5,348,761	ND	1.41
1-Penten-3-ol*	4.163 , 1.366	729	17,679,411 ± 829,055[a]	19,033,101 ± 439,252[a]	447,199 ± 63,363[b]	1.08
1-Propanol*	2.747 , 0.990	502	3,321,605 ± 36,836[b]	4,673,340 ± 163,461[a]	ND	1.13
2-Methyl-1-propanol*	3.330 , 1.155	590	20,904,555 ± 2,134,281[a]	13,254,444 ± 274,131[b]	ND	1.10
2-Butanol*	3.081 , 0.931	565	29,893,235 ± 3,382,785[a]	31,508,092 ± 1,114,167[a]	ND	1.00
3-Methoxy-2-butanol	10.908 , 2.825	936	67,779 ± 1,603	ND	ND	0.96
3-Methyl-2-butanol	4.163 , 1.056	729	254,989 ± 7,037[b]	5,399,032 ± 620,875[a]	ND	1.21
2-Buten-1-ol	3.913 , 1.782	721	ND	236,442 ± 12,493[b]	345,691 ± 38,440[a]	1.24
2-Furanmethanol*	9.076 , 0.825	880	ND	ND	34,776,532 ± 11,534,370	1.01
cis-5-Ethenyltetrahydro-5-trimethyl-2-furanmethanol	15.738 , 1.109	1083	56,623 ± 2,301	ND	ND	1.36
2-Heptanol*	10.158 , 1.214	913	2,729,513 ± 157,569[b]	3,527,832 ± 137,200[a]	ND	1.07
2-Hexanol*	6.994 , 1.247	816	115,701 ± 6,197[b]	7,231,740 ± 493,401[a]	6,666,314 ± 956,818[a]	1.18
(E)-2-Hexen-1-ol	8.993 , 1.960	878	112,789 ± 11,685	ND	ND	1.35
2-Morpholinomethyl-1,3-diphenyl-2-propanol	4.330 , 0.977	734	132,185 ± 9,646	ND	ND	0.98
2-Pentanol*	4.496 , 1.142	739	ND	14,968,216 ± 328,191	ND	1.20
2-Pentyn-1-ol	5.745 , 1.063	777	ND	ND	970,986 ± 286,867	1.01
1-Ethoxy-2-propanol	5.579 , 1.221	772	4,713,702 ± 1,631,718[a]	4,509,572 ± 722,097[a]	ND	0.90
1-Methoxy-2-propanol	3.996 , 1.267	724	14,447,466 ± 3,764,831[a]	906,949 ± 159,452[b]	ND	0.72
2-Propen-1-ol*	2.664 , 1.294	<500	1,411,206 ± 458,506[a]	480,483 ± 66,219[b]	1,331,831 ± 55,945[c]	1.15
3-Buten-1-ol	3.663 , 1.531	713	554,075 ± 79,954	ND	ND	1.35
3-Methyl-3-buten-1-ol	5.162 , 1.742	760	29,401 ± 3,069	ND	ND	0.94
2-Methyl-3-buten-2-ol	3.164 , 0.970	565	ND	1,121,410 ± 34,884[b]	24,238,592 ± 618,953[a]	1.07
3-Butyn-2-ol	5.412 , 1.452	767	ND	193,748 ± 3,061	ND	1.13
3-Hexen-1-ol	8.743 , 1.868	870	409,893 ± 48,587[a]	102,306 ± 4,267[b]	ND	1.26
3-Octanol*	13.323 , 1.122	1008	129,093 ± 44,125[a]	154,126 ± 34,090[a]	ND	1.01
3-Pentanol	4.496 , 1.089	739	7,441,664 ± 374,108[a]	7,017,034 ± 334,006[a]	ND	0.96
2-Methyl-3-pentanol	6.161 , 1.096	790	353,334 ± 7,921[a]	545,549 ± 121,666[a]	ND	1.10
3-Penten-2-ol	3.164 , 0.970	565	1,108,644 ± 22,141	ND	ND	1.18
4-Ethylcyclohexanol	14.655 , 1.478	1049	85,988 ± 14,091	ND	ND	1.20
4-Penten-1-ol	5.745 , 2.013	778	5,205,015 ± 249,293[b]	8,624,059 ± 132,945[a]	245,047 ± 13,790[c]	1.03

Cyclohexanol*	8.993 , 1.927	878	ND	165,788 ± 13,300	ND	1.03
Cyclopentanol*	6.245 , 1.987	793	772,962 ± 57,509[b]	94,146 ± 3,056[c]	1,254,826 ± 280,232[a]	0.96
2-Methyl-cyclopentanol	8.327 , 1.604	857	ND	422,034 ± 81,475	ND	1.09
3-Methyl-cyclopentanol	8.327 , 1.630	857	349,605 ± 59,306[a]	55,317 ± 22,575[b]	ND	1.08
6-Methyl-5-hepten-2-ol	12.906 , 1.063	995	3,208,628 ± 741,705[c]	4,824,846 ± 149,306[b]	7,974,278 ± 1,326,887[a]	0.64
Ethanol*	2.331 , 0.719	<500	ND	508,546 ± 96,891[b]	3,966,771 ± 95,883[a]	1.06
2-Ethoxy-ethanol*	4.829 , 1.670	736	466,997 ± 56,598[a]	433,474 ± 10,793[a]	ND	1.06
Isopropyl Alcohol*	2.414 , 0.713	<500	52,145,730 ± 169,798	ND	ND	1.36
Aldehydes						
1,2,3-Trimethyl-cyclopent-2-enecarboxaldehyde	13.573 , 0.627	1016	200,187 ± 7,941	ND	ND	0.96
1H-Pyrrole-2-carboxaldehyde	14.322 , 3.095	1040	ND	ND	4,080,601 ± 929,374	1.04
1-Ethyl-1H-pyrrole-2-carboxaldehyde	14.988 , 1.749	1060	ND	ND	316,964 ± 40,137	1.06
1-Methyl-1H-pyrrole-2-carboxaldehyde	13.573 , 2.178	1016	ND	ND	1,370,876 ± 77,233	1.07
(E,E)-2,4-Heptadienal*	13.323 , 1.432	1008	261,205 ± 65,512[b]	150,210 ± 38,960[c]	654,245 ± 57,128[a]	0.81
(E,E)-2,4-Hexadienal*	11.406 , 0.673	939	ND	3,255,017 ± 88,554	ND	1.03
2-Butenal*	3.663 , 0.957	640	957,314 ± 63,285[c]	3,959,736 ± ,1104,615[b]	20,478,644 ± 5,034,539[a]	0.71
(Z)-2-Butenal	3.996 , 0.904	723	ND	153,411 ± 4,176[b]	4,910,553 ± 118,460[a]	0.61
2-Ethyl-2-butenal	7.411 , 0.990	829	ND	ND	3,210,664 ± 138,295	0.91
(E)-2-Methyl-2-butenal	5.329 , 1.016	764	471,159 ± 99,435[c]	1,832,413 ± 365,937[b]	28,100,499 ± 1,363,246[a]	1.07
3-Methyl-2-butenal	5.329 , 1.010	764	151,952 ± 43,919	ND	ND	1.02
2-Ethyl-*trans*-2-butenal	7.411 , 0.997	829	157,556 ± 17,769[b]	251,576 ± 13,743[a]	ND	0.88
5-Methyl-2-furancarboxaldehyde	12.157 , 2.297	974	ND	92,209 ± 11,491[b]	61,104,039 ± 10,230,898[a]	1.05
(Z)-2-Heptenal*	11.990 , 1.168	968	800,233 ± 61,128[a]	110,597 ± 10,892[b]	ND	1.31
2-Hexenal*	8.576 , 1.096	864	399,164 ± 27,729[b]	959,070 ± 93,088[a]	1,162,732 ± 224,814[a]	1.17
2-Methyl-2-hexenal	9.242 , 0.904	885	ND	ND	315,698 ± 35,479	0.99
2-*n*-Butylacrolein	8.826 , 0.904	872	ND	61,032 ± 16,929	ND	1.36
(E)-2-Octenal*	15.321 , 1.082	1070	ND	279,204 ± 7,932[b]	511,115 ± 55,629[a]	0.89
(E)-2-Pentenal*	5.745 , 1.096	777	481,476 ± 35,164[b]	878,873 ± 40,232[a]	ND	1.18
2,2-Dimethyl-3,4-pentadienal	11.491 , 0.937	953	326,724 ± 92,199[a]	52,890 ± 11,299[b]	ND	0.82
3-Furaldehyde*	7.4110 , 2.653	829	ND	ND	1,544,063 ± 61,777	0.91
3-Methyl-2-thiophenecarboxaldehyde	16.154 , 2.099	1096	ND	ND	639,616 ± 65,102	1.07

4-Ethyl-2-hexynal	14.405 , 1.102	1041	ND	301,341 ± 6513[a]	308,014 ± 10,020[a]	0.76
(Z)-4-Heptenal	15.904 , 1.366	1088	149,340 ± 4,052	ND	ND	0.98
Acetaldehyde	2.165 , 1.531	<500	ND	348,907 ± 14,229[b]	10,853,730 ± 502,969[a]	0.88
Benzaldehyde*	12.157 , 1.921	973	418,449 ± 59,641[c]	872,741 ± 20,621[b]	4,878,889 ± 249,105[a]	1.07
Benzeneacetaldehyde*	14.822 , 1.967	1055	43,216 ± 1,510[b]	390,940 ± 17,549[b]	5,766,043 ± 614,072[a]	1.06
2-Methyl-butanal*	3.830 , 0.660	664	2,669,928 ± 395,165[c]	32,453,946 ± 1,291,557[b]	46,634,328 ± 2,015,653[a]	1.23
3-Methyl-butanal*	3.663 , 0.653	639	7,125,603 ± 154,861[c]	16,314,625 ± 339,020[a]	15,602,050 ± 427,150[b]	1.38
Furfural*	7.910 , 2.897	845	137,895 ± 14,837[b]	4,720,778 ± 56,888[b]	441,527,283 ± 17,955,165[a]	1.07
Heptanal*	10.075 , 0.851	910	10,372,295 ± 1,819,927[b]	13,209,653 ± 996,092[a]	341,680 ± 88,726[c]	1.09
Hexanal*	6.911 , 0.858	813	92,737,000 ± 10,063,198[a]	86,228,237 ± 1,077,068[a]	12,658,272 ± 684,576[b]	1.03
Methylglyoxal	3.164 , 2.581	699	ND	ND	659,413 ± 74,180	0.90
Nonanal*	16.737 , 0.878	1114	1,284,567 ± 105,677[b]	1,364,029 ± 305,176[b]	2,277,059 ± 696,631[a]	0.18
Octanal*	13.489 , 0.931	1013	1,736,248 ± 75,977[a]	1,493,247 ± 582,724[a]	ND	0.74
Pentanal*	4.330 , 0.772	734	33,163,256 ± 600,152[a]	34,775,709 ± 280,623[a]	17,021,782 ± 2,052,863[b]	1.07
2-Methyl-pentanal*	5.495 , 0.726	769	138,356 ± 10,836	ND	ND	0.88
2-Methyl-propanal*	2.747 , 0.561	685	ND	531,853 ± 8,831[b]	18,725,868 ± 1,653,616[a]	0.90
Aromatic hydrocarbons						
2,6-Bis(1,1-dimethylethyl)-4-(1-oxopropyl)phenol	27.728 , 0.746	1637	61,687 ± 17,009	ND	ND	1.31
2-Methoxy-4-vinylphenol*	22.816 , 3.392	1330	ND	ND	429,232 ± 25,032	0.93
1,2,3-Trimethyl-benzene*	13.156 , 0.904	1003	82,084 ± 2,911	ND	ND	0.96
1-Methyl-2-(1-methylethyl)-benzene*	14.322 , 0.739	1039	365,338 ± 69,059[b]	605,564 ± 133,493[a]	ND	0.65
Ethylbenzene*	8.743 , 0.851	870	165,126 ± 58,633[a]	27,013 ± 1,694[b]	ND	1.21
o-Xylene*	9.909 , 0.904	905	303,836 ± 176,630	ND	ND	0.90
Phenol	5.079 , 0.931	757	ND	ND	12,966,766 ± 4,732,058	0.87
p-Xylene*	8.993 , 0.858	877	692,576 ± 28,429[b]	355,660 ± 66,354[b]	4,960,792 ± 95,350[a]	0.68
Styrene*	9.742 , 1.115	900	305,146 ± 26,877[a]	124,141 ± 13,938[b]	ND	1.22
Toluene*	5.995 , 0.792	785	14,885,833 ± 1,323,029[b]	2,361,550 ± 481,430[c]	38,374,591 ± 17,939,113[a]	0.66
Esters						
1,2-Ethanediol, monoacetate	6.161 , 2.680	791	ND	ND	102,205,939 ± 450,579	0.82
1,3-Propanediol, diacetate	12.823 , 2.449	994	ND	ND	153,595 ± 4,783	0.92
2,2,4-Trimethyl-1,3-pentanediol diisobutyrate	23.732 , 1.274	1366	ND	415,397 ± 109,954	ND	0.96

(E)-2-Butenoic acid, methyl ester	5.912 , 0.957	782	ND	ND	1,380,694 ± 490,951	0.84
2-Furanmethanol, acetate*	13.156 , 1.696	1003	ND	ND	3,536,441 ± 156,329	1.07
2-Propenoic acid, 2-propenyl ester	8.993 , 1.630	878	ND	ND	276,745,183 ± 10,190,281	0.74
2-Propenoic acid, methyl ester	3.164 , 0.706	698	ND	ND	10,492,190 ± 1,828,072	1.05
(Z)-3-Hexen-1-ol, propanoate,	9.742 , 1.709	901	92,699 ± 1,745	ND	ND	0.95
3-Hydroxypyridine monoacetate	15.405 , 2.805	1073	ND	ND	772,580 ± 89,362	1.07
4-Pentenoic acid, methyl ester	7.161 , 0.924	821	ND	ND	1,568,402 ± 528,518	0.88
Acetic acid ethenyl ester	2.914 , 0.799	560	ND	ND	3,715,520 ± 100,774	0.93
Acetic acid, hydroxy-, methyl ester	4.496 , 3.808	740	ND	ND	1,194,417 ± 26,380	1.07
Acetic acid, methyl ester*	2.581 , 0.594	682	ND	6,122,693 ± 1,161,047[a]	7,655,912 ± 109,624[a]	0.73
Acetic acid, pentyl ester*	10.492 , 0.838	923	186,658 ± 32,840[b]	655,136 ± 43,466[a]	ND	0.73
Butanoic acid, 2-oxo-, methyl ester	7.911 , 1.538	844	ND	ND	1,122,021 ± 32,663	1.07
Butyrolactone*	10.492 , 3.617	924	8,339,108 ± 169,762[c]	14,636,027 ± 150,500[b]	73,176,295 ± 1,972,285[a]	1.07
Ethanol, 2-nitro-, nitrate (ester)	2.331 , 0.733	<500	5,261,548 ± 76,444[a]	5,516,956 ± 463,077[a]	ND	0.99
Formic acid, pentyl ester	7.744 , 0.911	839	390,345 ± 17,960[a]	365,654 ± 84,250[a]	ND	0.94
Hexanoic acid, methyl ester*	10.825 , 0.812	933	ND	784,492 ± 55,963[a]	603,662 ± 21,451[a]	0.79
Oxalic acid, diallyl ester	6.411 , 1.624	798	ND	ND	3,259,819 ± 305,252	0.44
Pantolactone	15.071 , 2.237	1063	ND	ND	2,533,374 ± 314,034	0.70
Propanoic acid, 2-hydroxy-, methyl ester	5.246 , 2.310	763	ND	849,961 ± 18,377[b]	6,063,687 ± 650,834[a]	0.57
Propanoic acid, 2-hydroxy-2-methyl-, methyl ester	7.244 , 2.039	824	ND	466,144 ± 63,757	ND	0.98
Propanoic acid, 2-methyl-, 2,2-dimethyl-1-(2-hydroxy-1-methylethyl)propyl ester	24.314 , 1.129	1390	129,155 ± 36,645[b]	353,043 ± 60,339[a]	ND	0.58
Propanoic acid, 2-methyl-, 3-hydroxy-2,4,4-trimethylpentyl ester	24.231 , 1.122	1387	304,632 ± 93,512[a]	266,147 ± 76,438[a]	ND	0.97
Propanoic acid, ethenyl ester	6.078 , 2.053	788	ND	2,026,786 ± 813,774	ND	0.96
S-Ethyl ethanethioate	8.243 , 1.617	854	ND	ND	902,686 ± 29,127	1.07
Triacetin*	23.481 , 1.888	1356	ND	415,242 ± 132,683[b]	1,194,963 ± 362,991[a]	0.87
Ethers						
2-Propenyl-3-vinyloxirane	5.246 , 0.680	762	ND	48,981 ± 937	ND	0.98
Dimethyl ether	2.248 , 0.726	<500	1,623,240 ± 50,435	ND	ND	0.91
[(1-Methylethoxy)methyl]-oxirane	10.908 , 2.798	936	ND	86,775 ± 478	ND	1.01
Hydrocarbons						

1,2,4,4-Tetramethylcyclopentene	8.660 , 0.541	867	ND	38,387 ± 9,329	ND	0.99
4,4-Dimethyl-1,2-pentadiene	4.413 , 0.700	736	1,887,080 ± 72,772	ND	ND	0.96
1,3,5-Cycloheptatriene	6.994 , 0.944	816	ND	ND	566,655 ± 174,207	0.87
1,3-Octadiene*	7.661 , 0.601	836	77,548 ± 20,506[a]	68,303 ± 16,532[a]	ND	0.87
3,3,6-Trimethyl-1,4-heptadiene	15.738 , 0.607	1082	ND	68,453 ± 11,607	ND	1.00
1,5-Dimethyl-6-oxa-bicyclo[3.1.0]hexane	7.327 , 0.660	826	ND	ND	133,546 ± 10,202	0.91
4,8-Dimethyl-1,7-nonadiene	15.738 , 0.620	1082	125,028 ± 21,480	ND	ND	1.12
1-Buten-3-yne	5.579 , 1.617	772	ND	ND	902,900 ± 78,374	0.75
1-Butene*	2.248 , 0.376	<500	ND	13,487,948 ± 321,876	ND	0.82
3,3-Dimethyl-1-butyne	10.741 , 1.247	930	ND	ND	33,271,440 ± 578,556	1.07
3,7,11-Trimethyl-1-dodecanol	14.655 , 0.561	1049	119,898 ± 42,419	ND	ND	0.89
1-Heptene*	4.163 , 0.455	728	47,394 ± 1,496[b]	510,720 ± 238,902[a]	ND	0.69
1-Nonene*	9.742 , 0.541	900	276,275 ± 57,173[a]	70,383 ± 18,845[b]	ND	1.14
1-Octene*	6.578 , 0.521	803	222,597 ± 33,244[a]	64,382 ± 14,162[b]	190,103 ± 8,265[a]	0.45
3,7-Dimethyl-1-octene	13.156 , 0.561	1003	ND	33,726 ± 567	ND	0.98
5-Nitro-1-pentene	12.490 , 1.445	983	ND	ND	4,309,544 ± 241,604	0.78
2-Methyl-1-propene*	2.414 , 0.370	<500	599,790 ± 190,685[a]	343,594 ± 44,741[b]	ND	0.75
3,3'-Thiobis-1-propene	8.660 , 0.898	867	66,651 ± 4,609	ND	ND	1.36
2,2,4,4-Tetramethyloctane	14.322 , 0.535	1039	349,162 ± 80,093[a]	61,253 ± 13,577[b]	ND	0.82
2,4,6,8-Tetramethyl-1-undecene	7.910 , 0.515	844	136,389 ± 18,247	ND	ND	1.16
2,4-Dimethyl-1-heptene	8.243 , 0.469	854	702,716 ± 392,571	ND	ND	0.80
2,6,6-Trimethyl-2-cyclohexene-1,4-dione	17.986 , 1.538	1156	ND	69,689 ± 3,291[b]	410,717 ± 39,180[a]	1.07
2,6-Octadiene	9.992 , 1.373	908	ND	272,110 ± 3,708	ND	1.05
5-Methyl-2-heptene	14.322 , 2.977	1040	ND	ND	19,966,34 ± 90,549	1.07
2-Methyl-1,5-heptadiene	9.409 , 1.591	890	ND	722,711 ± 9,490[b]	159,224,613 ± 2,821,947[a]	1.07
(Z)-5-Tridecene	14.905 , 0.581	1057	42,530 ± 13,394	ND	ND	0.83
1-Bromo-2-methyl-butane	16.404 , 1.419	1103	377,636 ± 86,772	ND	ND	0.95
2-Methyl-butane	2.747 , 0.436	500	20,435,973 ± 7,445,879	ND	ND	1.14
2-Nitro-butane	12.490 , 2.429	984	ND	3,650,089 ± 429,094[b]	9,402,987 ± 565,051[a]	0.66
(3-Methylpentyl)-cyclohexane	14.489 , 0.587	1044	ND	53,050 ± 224	ND	0.97
1-Ethyl-2-methyl-cyclohexane	10.325 , 0.541	918	34,998 ± 3,160	ND	ND	0.86

Butyl-cyclohexane	14.489 , 0.587	1044	253,596 ± 10,417	ND	ND	0.99
(S)-1-Methyl-4-(1-methylethenyl)-cyclohexene	14.322 , 0.726	1039	878,161 ± 171,877	ND	ND	0.82
1-Methyl-4-(1-methylethylidene)-cyclohexene	13.906 , 0.706	1026	57,873 ± 19,312	ND	ND	1.20
4-Bromo-cyclohexene	13.323 , 1.643	1008	229,743 ± 60,594	ND	ND	0.91
cis-1,2-Dimethyl-cyclopentane	4.246 , 0.436	731	ND	41,927 ± 11,595	ND	0.71
1-Methyl-3-(2-methyl-1-propenyl)-cyclopentane	13.239 , 0.620	1005	37,251 ± 845	ND	ND	0.77
1-Methyl-3-(2-methylpropyl)-cyclopentane	12.906 , 0.568	995	152,247 ± 36,069	ND	ND	0.89
Methyl-cyclopentane	5.995 , 0.891	785	1,674,036 ± 467,938	ND	ND	0.98
Propyl-cyclopropane	11.491 , 1.201	953	39,722 ± 6,294[b]	659,386 ± 57,466[a]	ND	0.68
Decane*	13.406 , 0.528	1010	277,539 ± 80,403[b]	81,144 ± 44,101[b]	1,166,057 ± 275,289[a]	0.57
2,3,5,8-Tetramethyl-decane	16.570 , 0.607	1109	187,535 ± 20,665	ND	ND	0.96
Heptane*	4.330 , 0.488	703	65,909 ± 11,981[b]	116,836 ± 37,789[b]	42,087,765 ± 4,949,993[a]	0.75
2,2,4,6,6-Pentamethyl-heptane	12.990 , 0.495	998	137,328 ± 29,626[a]	349,087 ± 87,048[a]	584,821 ± 291,369[a]	0.61
Hexane*	2.997 , 0.495	612	67,704,583 ± 782,343[a]	35,630,543 ± 6,752,750[b]	ND	1.12
1-Nitro-hexane	14.822 , 1.373	1054	133,570 ± 57,824[a]	160,256 ± 55,363[a]	ND	0.71
3-Ethyl-hexane	8.077 , 0.482	849	ND	148,675 ± 77,340	ND	0.73
3-Methyl-hexane	4.913 , 0.469	751	164,950 ± 39,732[a]	200,967 ± 22,748[a]	ND	0.57
Isopropylcyclobutane	4.163 , 0.917	729	ND	ND	780,283 ± 222,590	0.74
Nonane*	9.992 , 0.515	908	68,406 ± 3,095	ND	ND	0.96
Octane*	6.994 , 0.495	816	214,504 ± 42,058[b]	211,099 ± 135,326[b]	24,926,191 ± 344,717[a]	1.03
2,3,6-Trimethyl-octane	15.405 , 0.561	1072	127,984 ± 584	ND	ND	0.98
4-Methyl-octane	8.826 , 0.488	872	162,888 ± 41,249	ND	ND	0.95
Pentane*	2.414 , 0.442	500	13,864,969 ± 3,058,847[a]	10,268,184 ± 3,291,902[a]	524,401 ± 199,372[b]	1.00
Spiro[2.4]hepta-4,6-diene	6.245 , 0.858	7923	ND	138,112 ± 64,299	ND	0.95
Trichloromethane*	3.247 , 0.911	672	16,036,104 ± 451,878[a]	7,653,383 ± 194,082[b]	ND	1.16
Tridecane*	22.399 , 0.799	1311	175,835 ± 77,198[a]	215,020 ± 58,297[a]	ND	0.97
Undecane*	16.571 , 0.601	1109	ND	186,919 ± 58,830	ND	1.00
Isothiocyanates						
4-Isothiocyanato-1-butene	12.740 , 1.439	991	64,165,562 ± 16,522,583[a]	11,437,853 ± 175,440[b]	50,016,160 ± 1,098,335[a]	1.37
Allyl Isothiocyanate	9.492 , 1.518	893	4,303,070 ± 299,027[a]	832,267 ± 62,726[c]	2,365,459 ± 696,606[b]	1.38
2-Isothiocyanato-butane	10.991 , 1.010	938	1,206,577 ± 220,622[b]	1,388,445 ± 15,887[b]	10,276,934 ± 144,402[a]	1.07

Isothiocyanato-cyclopropane	8.993 , 1.096	877	89,962 ± 24,239	ND	ND	0.80
Isobutyl isothiocyanate	11.741 , 1.076	960	917,688 ± 166,134[b]	ND	30,436,416 ± 1,485,669[a]	0.70
Isopropyl isothiocyanate*	7.827 , 1.036	841	700,167 ± 249,087[b]	1,178,003 ± 224,967[b]	7,530,979 ± 1,937,342[a]	0.82
Isothiocyanato-methane*	5.246 , 1.643	762	ND	ND	1,600,250 ± 522,312	0.89
Ketones						
(*R*)-(+)-3-Methylcyclopentanone	8.327 , 1.089	857	40,562 ± 2,267[a]	52,950 ± 32,720[a]	ND	0.62
3-Methyl-1,2-cyclopentanedione	14.322 , 4.132	1040	ND	ND	717,066 ± 88,742	1.07
1-Hydroxy-2-butanone	6.078 , 2.772	788	770,649 ± 487,440[a]	ND	883,493 ± 18,729[a]	0.94
1-(2-Furanyl)-1-propanone	13.656 , 1.874	1019	ND	ND	902,945 ± 62,691	1.07
1-Cyclopropyl-1-propanone	5.995 , 0.871	785	ND	453,976 ± 119,125	ND	0.97
2,3-Pentanedione	12.074 , 1.914	971	ND	ND	18,320,431 ± 1,167,091	0.95
2,4-Pentanedione*	6.411 , 1.280	798	ND	ND	1,184,608 ± 58,826	0.75
2,5-Hexanedione*	10.991 , 2.092	938	ND	ND	698,229 ± 74,239	0.92
2-Butanone*	2.997 , 0.713	539	5,235,258 ± 59,456[c]	10,041,399 ± 4,465,526[b]	15,170,143 ± 1,855,453[a]	0.58
3-Hydroxy-2-butanone*	4.663 , 2.185	745	1,012,261 ± 78,369[c]	10,470,882 ± 2,640,944[b]	21,544,127 ± 893,835[a]	1.11
2-Cyclohexen-1-one*	11.158 , 1.630	943	ND	61,578 ± 5,342[b]	1,223,912 ± 1,9225[a]	1.07
3,5,5-Trimethyl-2-cyclohexen-1-one	15.321 , 0.997	1070	209,202 ± 69,425[b]	193,649 ± 75,985[b]	1,639,274 ± 104,352[a]	1.06
3-Methyl-2-cyclohexen-1-one	15.321 , 1.683	1070	ND	ND	273,103 ± 18,673	0.93
2-Cyclohexene-1,4-dione	14.239 , 3.010	1037	ND	ND	748,504 ± 54,031	1.07
2-Cyclopenten-1-one*	7.910 , 1.855	844	ND	552,717 ± 8,219[b]	4,888,188 ± 85,086[a]	1.07
2,3-Dimethyl-2-cyclopenten-1-one	14.655 , 1.511	1049	ND	54,840 ± 2,125[b]	213,700 ± 20,780[a]	1.08
2-Methyl-2-cyclopenten-1-one	10.242 , 1.465	916	56,447 ± 19,227[c]	323,040 ± 7,222[b]	4,141,721 ± 63,725[a]	1.07
3-Methyl-2-cyclopenten-1-one	4.663 , 0.686	744	ND	114,155 ± 5,324[b]	1,432,490 ± 368,374[a]	1.03
2-Cyclopentene-1,4-dione	9.492 , 3.670	894	ND	ND	2,112,366 ± 62,454	1.07
2-Decanone*	19.318 , 0.931	1200	ND	ND	399,642 ± 73,879	1.00
2-Heptanone*	9.742 , 0.891	900	4,798,263 ± 211,257[a]	2,913,389 ± 147,004[b]	1,263,506 ± 190,998[c]	1.16
2-Hexanone*	6.578 , 0.865	803	7,534,312 ± 209,873[a]	1,150,033 ± 235,900[c]	2,555,103 ± 67,025[b]	1.37
2-Hydroxy-3-pentanone	7.161 , 2.072	821	598,233 ± 25,358[c]	5,612,411 ± 91,152[b]	18,858,815 ± 264,950[a]	1.08
2-Nonanone*	16.237 , 0.917	1098	ND	103,486 ± 6,511[b]	3,230,794 ± 320,712[a]	0.96
2-Octanone*	13.073 , 0.904	1000	1,818,352 ± 633,028[b]	2,185,569 ± 690,007[b]	14,742,092 ± 1,154,970[a]	1.06
2-Pentanone*	4.163 , 0.752	728	1,559,768 ± 36,706[b]	1,920,280 ± 6,296[b]	29,525,013 ± 852,325[a]	0.96

3-Methyl-2-pentanone*	5.579 , 0.733	772	233,085 ± 146,777	ND	ND	1.00
1-(Acetyloxy)-2-propanone	8.993 , 2.482	878	ND	450,376 ± 5,435	ND	1.42
1-Hydroxy-2-propanone*	3.830 , 2.818	719	ND	715,431 ± 109,452[b]	18,705,933 ± 158,072[a]	0.82
(E,E)-3,5-Octadien-2-one	9.492 , 0.785	893	163,477 ± 61,118[a]	216,333 ± 81,842[a]	ND	0.95
3-Heptanone*	9.576 , 0.858	895	115,925 ± 45,256[a]	106,138 ± 3,875[a]	ND	0.89
3-Hexanone*	6.411 , 0.818	798	85,371 ± 32,119[b]	140,931 ± 9,431[a]	ND	1.07
3-Octanone*	12.906 , 0.878	995	65,821 ± 1,841[a]	95,020 ± 3,683[a]	ND	0.68
(E)-3-Octen-2-one*	14.655 , 1.129	1049	1,420,919 ± 222,038[b]	1,784,404 ± 56,230[b]	4,557,058 ± 1,179,172[a]	0.98
3-Pentanone	4.579 , 2.257	742	921,596 ± 129,419	ND	ND	0.75
2-Methyl-3-pentanone*	5.495 , 0.733	769	ND	1,025,310 ± 68,899	ND	1.41
3-Penten-2-one*	5.579 , 1.096	772	ND	2,474,101 ± 1,086,779[b]	13,458,316 ± 1,431,243[a]	1.05
3-Methyl-3-penten-2-one	8.077 , 1.102	849	ND	277,859 ± 12,509	ND	1.21
4-Methyl-3-penten-2-one	8.077 , 1.069	849	ND	ND	1,271,974 ± 408,445	1.01
4-(1-Hydroperoxy-2,2-dimethyl-6-methylene-cyclohexyl)-pent-3-en-2-one	25.730 , 1.175	1475	85,973 ± 26,054	ND	ND	0.94
4-Hydroxy-3-hexanone	9.992 , 1.703	908	ND	ND	323,002 ± 22,595	1.07
6-Methyl-5-hepten-2-one*	12.906 , 1.010	995	7,857,126 ± 947,223[c]	12,765,914 ± 1,554,109[b]	31,323,551 ± 952,859[a]	1.07
Acetone*	2.414 , 0.554	<500	12,956,207 ± 214,727[c]	19,584,661 ± 219,120[b]	26,145,548 ± 701,423[a]	1.15
Acetophenone*	15.571 , 1.868	1078	1,452,722 ± 6,246[a]	ND	288,045 ± 18,024[b]	0.78
Cyclohexanone*	9.909 , 1.214	905	28,545 ± 3,092	ND	ND	1.35
2,2,6-Trimethyl-cyclohexanone*	14.572 , 0.904	1047	78,080 ± 7,181[b]	121,417 ± 14,558[b]	283,675 ± 106,324[a]	0.85
3-Butyl-cyclohexanone	16.154 , 0.594	1095	68,137 ± 12,057	ND	ND	1.04
4-Ethyl-cyclohexanone	13.656 , 1.023	1018	90,352 ± 5,240[a]	193,965 ± 93,063[a]	ND	0.82
Cyclopentanone*	6.661 , 1.201	806	140,473 ± 4,502[c]	295,356 ± 2,506[b]	366,700 ± 70,253[a]	1.15
2-Methyl-cyclopentanone*	8.160 , 1.063	852	473,112 ± 52,019[a]	657,743 ± 84,320[a]	ND	1.11
3-Methyl-cyclopentanone*	8.410 , 1.129	859	101,459 ± 16,764[a]	81,532 ± 1,912[a]	ND	0.77
Dimethylketene	14.738 , 3.016	1053	ND	ND	4,080,770 ± 442,824	0.75
1-(1H-Pyrrol-2-yl)-Ethanone	15.738 , 0.363	1082	ND	ND	2,959,330 ± 118,884	0.98
1-(2-Furanyl)-Ethanone	10.408 , 2.270	921	ND	356,167 ± 7,735[b]	54,160,114 ± 2,027,417[a]	1.07
1-(2-Pyridinyl)-Ethanone	14.489 , 1.848	1044	ND	ND	395,329 ± 60,218	1.06
1-(2-Thienyl)-Ethanone	16.320 , 2.468	1101	ND	ND	555,683 ± 75,641	1.06
1-(4,5-Dihydro-2-thiazolyl)-Ethanone	16.737 , 2.171	1115	ND	ND	749,390 ± 83,141	1.07

Nitrogen-containing compounds						
(3*R*)-(+)-3-Acetamidopyrrolidine	8.826 , 1.907	873	ND	701,840 ± 21,547	ND	1.41
1H-1,2,4-Triazole	8.993 , 1.934	878	ND	ND	3,548,579 ± 1,381,276	0.72
2,3-Dimethyl-5-ethylpyrazine*	15.904 , 1.082	1088	ND	ND	1,687,443 ± 42,873	0.85
2,4-Dihydroxybenzamidine	17.070 , 1.307	1126	ND	ND	570,580 ± 48,254	1.07
2-Acetyl-1,4,5,6-tetrahydropyridine*	15.821 , 1.597	1085	ND	ND	1,180,049 ± 175,352	1.06
2-Acetyl-3-methylpyrazine*	17.070 , 1.742	1126	ND	ND	763,139 ± 207,712	1.03
cis- tetrahydro-3α,6α-dimethyl 2H-Furo[2,3-b]pyrrole-2,5(3H)-dione	18.652 , 2.264	1179	ND	ND	1,128,762 ± 180,395	1.06
5,6-Dihydro-2H-pyran-2-one,	14.489 , 4.336	1046	ND	ND	2,626,989 ± 676,053	1.03
Tetrahydro-6-methyl-2H-pyran-2-one,	16.404 , 2.620	1104	ND	ND	17,522,824 ± 126,712	0.50
2-Isobutyl-3-methylpyrazine	17.736 , 1.003	1148	ND	ND	460,073 ± 36,525	1.07
1-Methyl-2-pyrrolidinone*	15.238 , 2.119	1068	ND	ND	1,227,102 ± 164,540	1.06
2-(1-Hydroxy-1-methyl-2-oxopropyl)-2,5-dimethyl-3(2H)-furanone	11.741 , 1.848	961	ND	ND	310,686 ± 17,363	1.07
Dihydro-2-methyl-3(2H)-furanone*	7.077 , 1.439	819	ND	438274 ± 21069[b]	11,869,480 ± 1,360,911[a]	1.06
3-Acetyl-1-methylpyrrole	15.821 , 1.802	1085	ND	ND	139,664 ± 7,651	0.77
3-Methylpyridazine	9.576 , 1.861	896	ND	ND	703,248 ± 14,652	0.63
N-Acetyl-4(H)-pyridine,	13.989 , 2.871	1030	ND	ND	3,229,618 ± 51,862	0.78
4-Methyl-5H-furan-2-one	14.822 , 4.613	1056	ND	263720 ± 23760[b]	1,484,672 ± 99,137[a]	1.07
5H-5-Methyl-6,7-dihydrocyclopentapyrazine	17.903 , 1.313	1153	ND	ND	988,659 ± 117,075	1.07
Acetylpyrazine*	14.155 , 2.079	1034	ND	ND	1,137,952 ± 386,773	1.01
Diethylcyanamide	9.076 , 2.112	880	ND	ND	878,235 ± 62,063	1.07
N,N-Dimethyl-formamide,	7.660 , 1.848	837	295,215 ± 9,792[a]	328,539 ± 14,129[a]	ND	1.08
N-Methyl-formamide	4.413 , 1.096	736	ND	310,982 ± 88,007	ND	0.56
1-Methyl-1-(2-methylpropyl)-hydrazine	14.655 , 1.795	1050	ND	61,199 ± 1,373	ND	1.03
O-(2-Methylpropyl)-hydroxylamine	5.745 , 0.436	777	45,862 ± 14,178	ND	ND	0.85
N-Methoxy-methanamine	2.747 , 1.544	504	57,286 ± 9,035[b]	144,314 ± 5,607[a]	ND	1.02
N-Vinylimidazole	8.660 , 1.214	867	ND	ND	128,681 ± 6,251	0.76
Pyrazine*	5.246 , 1.452	762	ND	ND	2,6821,770 ± 656,991	1.07
(1-Methylethenyl)-pyrazine	14.072 , 1.353	1031	ND	ND	1,903,058 ± 174,151	1.07
2,5-Dimethyl-pyrazine*	10.492 , 1.221	923	281,604 ± 64,434[c]	5,930,421 ± 126,518[b]	244,702,298 ± 3,902,254[a]	1.07

2,5-Dimethyl-3-propyl-pyrazine	18.402 , 0.950	1170	ND	ND	275,710 ± 5,814	0.77
2-Ethenyl-5-methyl-pyrazine	14.155 , 1.373	1034	ND	ND	1,111,549 ± 97,143	1.07
2-Ethyl-3-methyl-pyrazine*	13.573 , 1.115	1016	ND	ND	66,047,374 ± 1,839,513	1.07
2-Ethyl-6-methyl-pyrazine*	13.406 , 1.089	1011	ND	ND	25,670,999 ± 885,224	0.90
2-Methoxy-3-(1-methylethyl)-pyrazine	16.237 , 0.983	1098	17,950 ± 1,070	ND	ND	1.36
(E)-2-Methyl-5-(1-propenyl)-pyrazine	16.570 , 1.195	1109	ND	ND	162,640 ± 9,777	0.56
2-Methyl-5-propyl-pyrazine	16.320 , 1.043	1100	ND	ND	736,541 ± 275,207	1.00
3-Ethyl-2,5-dimethyl-pyrazine*	15.904 , 1.023	1088	ND	533,262 ± 92,236[b]	10,640,034 ± 880,494[a]	1.07
Ethenyl-pyrazine	11.158 , 1.597	943	ND	ND	817,633 ± 43,796	1.07
Ethyl-pyrazine*	10.658 , 1.241	928	ND	ND	19,109,505 ± 2,508,783	1.06
Isopropenyl-pyrazine*	15.904 , 1.525	1088	ND	ND	174,100 ± 11,431	0.63
Methyl-pyrazine*	7.660 , 1.353	836	161,811 ± 7,512[b]	3,343,461 ± 40,085[b]	236,948,508 ± 3,893,685[a]	1.07
Trimethyl-pyrazine*	13.739 , 1.102	1021	ND	520937 ± 63,120	ND	1.40
Pyridine*	5.7456 , 1.228	777	794,448 ± 184,868[b]	1,754,645 ± 218,613[b]	14,810,098 ± 256,596[a]	1.07
2-Methyl-pyridine*	7.577 , 1.148	834	ND	ND	1,622,161 ± 311,889	1.05
Pyrrole*	5.828 , 0.495	780	321,248 ± 21,085[b]	356,094 ± 29,717[b]	11,535,292 ± 595,770[a]	1.07
Tetrahydrofurfuryl acrylate	10.575 , 1.346	926	ND	ND	1,451,339 ± 36,284	0.76
8-Methyl-tetrazolo[1,5-b]pyridazine	8.160 , 2.759	853	ND	68,999 ± 580	ND	1.02
Nitriles						
(E)-2-Pentenenitrile	6.994 , 1.643	816	ND	ND	853,510 ± 91,648	0.87
2,4-Hexadienenitrile	11.074 , 1.993	941	ND	66,773 ± 10,894[b]	12,626,949 ± 810,475[a]	1.07
2,4-Pentadienenitrile	6.411 , 2.006	798	ND	21,717,723 ± 338,100[b]	179,300,812 ± 2,703,074[a]	1.07
2-Butenenitrile*	4.080 , 1.373	726	ND	ND	57,776,201 ± 2,257,276	1.07
2-Furancarbonitrile	6.911 , 2.442	814	ND	ND	816,170 ± 18,366	1.07
2-Pentenenitrile	5.828 , 1.802	780	ND	ND	1,989,594 ± 241,331	0.46
2-Propenenitrile	2.581 , 0.851	<500	145,899 ± 71,953[b]	ND	5,100,494 ± 269,294[a]	0.87
2-Methyl-2-propenenitrile	3.580 , 1.162	711	ND	943,330 ± 141,040	ND	1.39
3-Butenenitrile*	3.830 , 1.571	719	184,909 ± 25,879[b]	16,115,886 ± 141,356[a]	ND	1.35
3-Pentenenitrile	5.828 , 1.822	780	71,819,412 ± 2,119,879[b]	126,077,229 ± 872,484[b]	383,964,323 ± 74,863,154[a]	0.78
2-Methyl-5-hexenenitrile	8.743 , 1.650	870	3,154,080 ± 116,485[c]	177,043,789 ± 1,308,301[a]	83,867,185 ± 38,480,183[b]	0.98
Acetonitrile*	2.414 , 0.891	<500	ND	7,739,211 ± 694,748[b]	25,532,397 ± 2,722,743[a]	0.65

Azeleonitrile	14.822 , 1.168	1054	ND	ND	6,200,035 ± 452,295	1.07
Benzenepropanenitrile*	20.734 , 2.878	1252	ND	ND	9,583,337 ± 1,659,157	1.06
Benzonitrile*	12.906 , 2.284	996	ND	ND	243,427 ± 17,146	1.07
Benzyl nitrile*	17.736 , 3.208	1149	ND	ND	6,626,368 ± 967,232	1.06
2-Methyl-butanenitrile	4.912 , 0.997	752	ND	15,416,744 ± 173,008[b]	64,651,388 ± 3,002,649[a]	1.08
3-Methyl-butanenitrile	5.079 , 1.109	757	ND	11,140,643 ± 2,269,189[b]	25,036,326 ± 1,240,067[a]	1.12
4-(Methylthio)-butanenitrile	16.071 , 2.627	1094	ND	ND	1,534,809 ± 136,892	1.07
1-Methyl-2-(1-methylethenyl)-cyclobutaneacetonitrile	6.828 , 1.716	811	ND	165,574 ± 13,133	ND	1.00
3,3-Dimethyl-cyclobutanecarbonitrile	10.075 , 1.023	910	ND	ND	1,655,333 ± 52,732	0.76
Isocyanato-cyclohexane	13.323 , 0.891	1008	47,971 ± 14,783[a]	52,909 ± 3,355[a]	ND	0.67
Heptanonitrile*	12.657 , 1.247	988	ND	ND	24,164,998 ± 1,013,173	1.07
Hexanenitrile	11.741 , 1.214	960	ND	2,959,832 ± 50,735[b]	12,638,654 ± 177,115[a]	1.08
5-Methyl-hexanenitrile	11.574 , 1.214	955	ND	2,012,774 ± 33,899[b]	49,283,746 ± 920,250[a]	0.93
Isobutyronitrile*	3.247 , 0.851	672	ND	1,226,640 ± 23,713[b]	6,981,368 ± 875,390[a]	0.88
Methallyl cyanide	5.745 , 1.782	778	ND	1,173,535 ± 92,932[b]	12,768,083 ± 3,772,205[a]	0.86
Methyl isocyanide	2.414 , 0.878	<500	ND	ND	19,888,461 ± 1,211,463	0.67
4,4-Dimethyl-5-oxo-pentanenitrile	15.071 , 1.195	1062	ND	ND	1,296,115 ± 410,536	1.02
4-Methyl-pentanenitrile	8.160 , 1.208	852	ND	273,934 ± 20,665[b]	7,612,592 ± 860,376[a]	1.06
5-(Methylthio)-pentanenitrile	19.568 , 2.488	1210	ND	ND	4,313,088 ± 634,362	1.06
O-heterocyclic compounds						
4,7-Dihydro-1,3-dioxepin	14.572 , 3.788	1048	ND	ND	27,916,227 ± 1,466,220	0.75
1,4-Dioxane	4.663 , 0.937	744	898,112 ± 42,146	ND	ND	0.96
5-Ethenyldihydro-5-methyl-2(3H)-furanone	14.655 , 2.323	1050	52,382 ± 3,625[a]	67,008 ± 4,474[a]	ND	1.10
5-Ethyldihydro-2(3H)-furanone	15.155 , 2.534	1065	2,272,447 ± 132,352[b]	2,729,378 ± 97,723[b]	4,186,532 ± 184,012[a]	1.07
Dihydro-3,5-dimethyl-2(3H)-furanone	13.489 , 2.191	1014	ND	223,068 ± 51,877	ND	1.15
Dihydro-3-methyl-2(3H)-furanone	11.657 , 2.660	959	576,234 ± 18,163[a]	746,358 ± 10,068[a]	ND	1.11
Dihydro-4,5-dimethyl-2(3H)-furanone	13.489 , 2.218	1014	210,331 ± 32,412	ND	ND	1.34
Dihydro-4-methyl-2(3H)-furanone	12.074 , 2.772	971	629,390 ± 23,614[a]	856,660 ± 15,252[a]	368,390 ± 13,020[b]	0.71
Dihydro-5-methyl-2(3H)-furanone*	11.824 , 2.851	964	2,055,071 ± 65,164[b]	2,572,144 ± 40,761[a]	2,137,857 ± 36,935[b]	1.43
Dihydro-5-propyl-2(3H)-furanone	18.152 , 2.132	1162	ND	150,784 ± 9,236[b]	417,268 ± 44,000[a]	1.10
2(5H)-Furanone	6.245 , 2.845	794	ND	130,081 ± 49,827[b]	6,690,878 ± 559,222[a]	0.72

3-Methyl-2(5H)-furanone*	12.573 , 3.762	987	ND	12,9580 ± 5,263[b]	665,252 ± 98,339[a]	0.71
5,5-Dimethyl-2(5H)-furanone	11.741 , 2.818	961	55,534 ± 10,750[b]	157,714 ± 6,633[a]	ND	1.28
5-Ethyl-2(5H)-furanone	14.572 , 3.161	1048	ND	217,727 ± 16,090[b]	1,018,858 ± 110,979[a]	1.07
5-Methyl-2(5H)-furanone*	11.324 , 3.973	949	ND	116,656 ± 27,784[b]	4,822,360 ± 72,364[a]	1.07
3,4-Dimethyl-2,5-furandione	14.405 , 2.884	1042	ND	ND	271,040 ± 10,225	0.76
Dihydro-3-methyl-2,5-furandione	14.572 , 3.762	1048	ND	ND	13,126,685 ± 2,778,631	0.76
2-Acetyl-5-methylfuran*	14.489 , 1.947	1044	ND	ND	280,389 ± 10,024	0.93
2,5-Dihydro-3,5-dimethyl 2-furanone	13.323 , 2.521	1009	ND	ND	431,905 ± 37,226	1.07
2-*n*-Butyl furan*	9.742 , 0.792	900	469,026 ± 21,467[b]	600,106 ± 54,370[b]	2,280,238 ± 278,208[a]	0.85
Furan	2.414 , 0.495	<500	ND	ND	9,949,222 ± 345,930	0.91
2-(2-Propenyl)-furan	8.576 , 1.049	865	ND	ND	772,934 ± 272,439	1.01
2,2'-[Oxybis(methylene)]bis-furan	22.399 , 2.006	1312	ND	ND	226,834 ± 12,238	1.07
2,2'-Methylenebis-furan	15.987 , 1.538	1090	ND	ND	148,297 ± 4,552	0.76
2,5-Dimethyl-furan*	4.496 , 0.673	739	ND	ND	1,120,566 ± 438,209	0.70
2-[(Methyldithio)methyl]-furan	19.984 , 1.683	1224	ND	ND	326,878 ± 41,260	1.07
2-[(Methylthio)methyl]-furan	13.323 , 1.445	1008	ND	ND	2,076,846 ± 518,432	0.72
2-Ethyl-furan*	4.413 , 0.693	736	ND	ND	4,253,760 ± 26,759	0.78
2-Methoxy-furan	5.495 , 1.274	770	ND	54,883 ± 12,573[b]	631,281 ± 287,634[a]	0.76
2-Methyl-furan	3.080 , 0.587	695	ND	ND	27,056,837 ± 2,862,072	1.06
2-Pentyl-furan*	13.073 , 0.785	1000	261,662 ± 9,760[b]	354,253 ± 106,143[b]	1,125,803 ± 97,732[a]	1.02
2-Propyl-furan*	6.495 , 0.766	800	144,009 ± 10,448[a]	197,113 ± 80,583[a]	ND	0.69
3-Methyl-furan	5.662 , 1.525	775	45,228 ± 16,263	ND	ND	0.55
Furfuryl formate	10.242 , 2.237	916	ND	ND	1,339,820 ± 156,481	1.07
4,5-Dimethyl-oxazole	5.912 , 1.082	782	ND	ND	1,526,206 ± 125,391	1.07
Trimethyl-oxazole	8.493 , 0.983	862	ND	ND	1,532,902 ± 105,153	1.07
Sulfur-containing compounds						
[1,4]Dithian-2-one	15.821 , 1.894	1085	ND	ND	1,085,355 ± 90,449	1.07
1,2,3-Trithiolane	17.236 , 2.165	1132	ND	ND	164,550 ± 14,465	1.07
2,4-Dithiapentane*	9.742 , 1.214	900	271,408 ± 20,024[a]	83,874 ± 6,677[b]	ND	1.25
2-Acetylthiazole*	13.989 , 2.231	1029	ND	ND	363,172 ± 3,4179	1.07
Dihydro-2-methyl-3(2H)-thiophenone	13.073 , 1.709	1001	ND	ND	2,591,224 ± 97,683	1.07

3-Thiophenecarboxaldehyde	13.489 , 2.838	1014	ND	ND	3,849,509 ± 89,761	0.95
4-Ethyl-5-methylthiazole*	14.572 , 1.234	1047	ND	ND	270,022 ± 54,535	1.05
4-Methylthiazole*	7.494 , 1.439	831	ND	ND	10,215,096 ± 1,569,757	1.06
5-Ethylthiazole*	11.824 , 1.340	963	ND	ND	443,976 ± 80,974	1.06
8-Thiabicyclo[3.2.1]octane	18.485 , 2.224	1173	ND	62,059 ± 3,689[b]	455,519 ± 20,926[a]	1.07
Carbon disulfide	2.581 , 0.488	<500	ND	2,964,201 ± 909,062[b]	37,982,378 ± 15,551,474[a]	0.93
Carbonyl sulfide	1.998 , 4.382	<500	2,340,158 ± 706,477	ND	1,718,617 ± 178,222	0.71
Diallyl carbonate	8.077 , 1.208	849	ND	146,612 ± 6,221	ND	0.99
Dihydro-2-(3H)-thiophenone	13.406 , 2.396	1011	ND	ND	425,653 ± 17,393	1.07
Dihydro-3-(2H)-thiophenone	11.824 , 2.244	963	ND	144,524 ± 6,734[b]	4,070,900 ± 64,534[a]	1.07
Dimethyl sulfide	2.498 , 0.502	<500	105,392,515 ± ,2482,879[a]	74,685,145 ± 3,494,206[b]	30,412,840 ± 5,553,485[c]	1.13
Dimethyl sulfone*	11.074 , 4.231	942	10,637,225 ± 1,174,631[b]	21,205,574 ± 276,990[a]	4,570,633 ± 1,440,764[c]	1.27
Dimethyl Sulfoxide*	8.660 , 3.683	868	290,638,933 ± 81,513,660[a]	316,258,806 ± 2,350,627[a]	309,384,904 ± 4,260,380[a]	0.35
Dimethyl trisulfide*	12.407 , 1.208	980	ND	628,373 ± 18,214[b]	34,862,642 ± 650,966[a]	1.07
Dimethyl disulfide*	5.329 , 0.924	764	1,814,991 ± 645,395[b]	88,643,327 ± 757,167[a]	98,276,228 ± 19,209,568[a]	1.29
Methyl (methylthio)methyl disulfide	17.486 , 1.518	1140	ND	187,188 ± 9,289[b]	2,276,570 ± 183,169[a]	1.07
2-(Methylthio)-ethanol*	8.160 , 3.755	853	99,435 ± 36,099	ND	ND	1.27
Methanethiol*	2.498 , 1.756	<500	772,290 ± 264,995[b]	5,107,378 ± 1,510,791[a]	5,182,353 ± 1,326,508[a]	1.13
Methyl thiolacetate	9.242 , 1.624	885	ND	ND	738,846 ± 29,250	1.07
Monomethyl carbonotrithioate	3.247 , 0.924	700	ND	ND	5,584,331 ± 710,575	0.94
3-(Methylthio)-propanal*	10.325 , 1.907	918	ND	66,260 ± 17,877[b]	360,421 ± 21,997[a]	1.07
1-(Methylthio)-propane	5.745 , 1.703	778	ND	707,485 ± 183,970	ND	1.15
Sulfur dioxide	3.247 , 1.709	701	1,172,407 ± 77,306[b]	144,143 ± 25,196[c]	2,016,350 ± 92,825[a]	0.95
Thiazole*	5.412 , 1.683	767	807,585 ± 18,264[c]	3,334,177 ± 24,780[b]	12,465,011 ± 549,505[a]	1.07
2,4-Dimethyl-thiazole*	9.659 , 1.155	898	ND	ND	1,185,706 ± 432,425	0.98
2-Ethyl-thiazole*	9.992 , 1.188	908	ND	ND	212,702 ± 4,334	0.77
2-Methyl-thiazole	7.244 , 1.274	824	97,523 ± 26,145[c]	668,008 ± 120,304[b]	3,958,261 ± 138,511[b]	1.07
4,5-Dimethyl-thiazole*	11.241 , 1.294	946	ND	ND	492,640 ± 142,714	1.03
4-Ethyl-2-methyl-thiazole	12.573 , 1.076	985	ND	ND	419,546 ± 17,841	1.07
5-Ethyl-2-methyl-thiazole	13.989 , 1.129	1029	ND	ND	265605 ± 51925	0.90
5-Methyl-thiazole	8.160 , 1.432	852	ND	ND	320037 ± 4937	1.07

Thiocyanic acid, methyl ester	4.579 , 2.086	742	2,498,878 ± 75,570[b]	7,395,999 ± 57,460[b]	61,227,781 ± 1,679,866[a]	1.07
Thiophene	3.913 , 0.865	721	ND	2,553,325 ± 18,074[b]	42,085,346 ± 2,048,162[a]	1.07
2,3-Dimethyl-thiophene*	9.326 , 0.944	888	ND	ND	282,809 ± 9,823	0.75
2-Ethyl-thiophene*	8.909 , 0.924	875	ND	ND	784,379 ± 172,756	0.74
2-Methyl-thiophene*	6.078 , 0.904	788	ND	19,6559 ± 24,916[b]	2,440,357 ± 662,438[a]	0.74
3-(Methylthio)-thiophene	16.071 , 1.729	1093	ND	ND	371,128 ± 31,047	1.07
3-Methyl-thiophene*	6.245 , 0.904	793	ND	187,337 ± 33,712[b]	4,688,512 ± 247,218[a]	0.86
Terpenes						
(+)-4-Carene*	13.239 , 0.693	1005	15,967 ± 1,768	ND	ND	1.17
1,3,8-*p*-Menthatriene	14.489 , 0.818	1044	86,310 ± 1,352	ND	ND	0.83
5-(1,1-Dimethylethyl)-1,3-cyclopentadiene	8.160 , 0.587	851	ND	39,838 ± 10,071	ND	1.17
(1S)-1,5-Dimethyl-6,8-Dioxabicyclo[3.2.1]octane	11.824 , 0.838	963	21,348 ± 509[b]	242,252 ± 74,481[a]	ND	1.09
α-Myrcene*	12.990 , 0.706	998	140,855 ± 34,648[b]	186,698 ± 34,984[b]	519,352 ± 169,788[a]	0.75
α-Phellandrene*	15.321 , 0.733	1070	53,004 ± 14,413	ND	ND	1.06
α-Pinene*	12.657 , 0.640	988	324,330 ± 25,028[a]	ND	108,479 ± 9,721[b]	0.92
(1α,2α,5α)-5-Methyl-bicyclo[3.1.0]hexan-2-ol	14.489 , 0.713	1044	82,113 ± 7,174	ND	ND	0.90
Bicyclo[3.1.0]hexan-3-one	9.076 , 1.340	880	ND	61,022 ± 1,525	ND	1.21
4-Methyl-1-(1-methylethyl)-bicyclo[3.1.0]hexane, didehydro deriv.	10.908 , 0.601	935	465,166 ± 137,315[a]	319,278 ± 4,107[a]	157,196 ± 10,216[a]	0.88
4-Methylene-1-(1-methylethyl)-bicyclo[3.1.0]hexane	12.490 , 0.680	983	105,379 ± 42,822	ND	ND	1.23
2,6,6-Trimethyl-bicyclo[3.1.1]hept-2-ene	11.158 , 0.568	943	912,161 ± 58,646[a]	804,584 ± 59,704[a]	1,658,273 ± 791,287[a]	0.64
(1α,2α,5α)- 2,6,6-Trimethyl-, Bicyclo[3.1.1]heptan-3-one	13.739 , 0.647	1021	30,445 ± 9,184	ND	ND	0.96
Camphene*	11.741 , 0.620	960	125,959 ± 19,732	58077 ± 424	ND	1.17

Different letters in the same row indicate a significant difference ($p < 0.05$) (LSD Fisher, ANOVA).

([1]) RT – retention time; ([2]) LRI – linear retention index. Compounds marked with * had retention indices within 3% of values reported in NIST library

Supplementary Table 2. Volatile compounds tentatively identified by GC×GC-ToFMS in press cakes obtained from roasted seeds. Variable Importance in Projection (VIP) scores for each compounds informs about the contribution to the separation between the studies oils. Results expressed and mean of peak areas of 5 replicates ± SD.

	RT[1] (min)	LRI[2]	Control	140 °C	180 °C	VIP
Acids						
Acetic acid*	3.330 , 2.178	617	ND	ND	573,009,903 ± 145,491,593	1.17
(Acetyloxy)-acetic acid	6.078 , 2.732	788	ND	634,398 ± 23,223	ND	1.01
Acetic anhydride	6.661 , 3.571	807	ND	198,399 ± 66,442[b]	23,980,282 ± 5,922,458[a]	1.03
Methyltartronic acid	4.663 , 2.204	726	ND	824,367 ± 87,884	ND	0.94
Alcohols						
1,5-Hexadien-3-ol	12.99 , 0.898	998	617,465 ± 272,510[b]	ND	152,194,610 ± 43,884,988[a]	1.13
3,7-Dimethyl-1,6-octadien-3-ol*	16.571 , 1.234	1109	107,616 ± 50,683[a]	102,962 ± 32,923[a]	ND	0.96
1-Butanol*	3.830 , 1.353	678	3,386,842 ± 541,434[a]	1,151,166 ± 125,156[b]	1,102,716 ± 75,887[b]	1.35
2-methyl-1-butanol*	5.329 , 1.426	765	254,665 ± 110,823[a]	188,153 ± 26,190[a]	ND	0.92
3-Methyl-1-butanol*	5.246 , 1.452	762	662,525 ± 85,645	ND	ND	1.02
2-Ethyl-1-decanol	13.739 , 0.554	1021	ND	ND	340,967 ± 130,986	0.91
2-Hexyl-1-decanol	26.313, 0.554	1514	ND	442,245 ± 93,552	ND	0.98
1-Heptanol*	12.491 , 1.439	983	895,308 ± 89,178[a]	688,194 ± 18,306[a]	ND	1.29
1-Hexanol*	9.159 , 1.518	883	ND	6,447,160 ± 243,840[a]	3,121,281 ± 49,611[b]	1.39
2-Ethyl-1-hexanol*	12.491 , 1.439	983	1,357,190 ± 380,933[a]	1,176,743 ± 152,203[a]	1,186,941 ± 195,272[a]	0.85
1-Nonanol*	18.986 , 1.234	1189	255,579 ± 15,181	ND	ND	1.15
2,7-Dimethyl-1-octanol	14.905 , 0.541	1057	70,252 ± 16,821	ND	ND	0.79
2-Butyl-1-octanol	14.905 , 0.561	1057	59,801 ± 4,206[c]	135,070 ± 45,949[b]	542,591 ± 140,023[a]	0.63
1-Pentanol*	6.079 , 1.558	788	604,047 ± 382,374[a]	217,844 ± 7,612[b]	168,027 ± 62,349[b]	0.90
4-Methyl-1-pentanol	9.159 , 1.544	887	6,521,371 ± 635,971	ND	ND	0.95
2-(2-Hydroxypropoxy)-1-propanol	20.734 , 1.082	1251	56,292 ± 17,870[a]	50,362 ± 3,226[a]	ND	0.84
2,4,7,9-Tetramethyl-5-decyn-4,7-diol	24.897 , 1.221	1422	395,895 ± 82,455[a]	365,621 ± 33,521[a]	ND	1.15
2-Buten-1-ol	3.914 , 1.782	721	889,447 ± 91,257	ND	ND	1.15
2-Methyl-2-buten-1-ol	6.245 , 1.973	793	319,174 ± 31,820	ND	ND	1.18
2-Furanmethanol*	9.076 , 0.825	880	ND	ND	4,701,164 ± 1,596,836	1.16

2-Hexanol*	6.995 , 1.247	816	1,892,178 ± 186,948[b]	220,278 ± 2,517[b]	75,344,648 ± 3,593,702[a]	1.20
(E)-2-Hexen-1-ol	8.993 , 1.960	878	ND	128,824 ± 14,108	ND	1.41
2-Hexyl-1-octanol	24.981 , 0.528	1427	80,114 ± 22,830	ND	ND	0.54
2-Methyl-1-undecanol	20.901 , 0.568	1256	350,698 ± 32,831[a]	279,556 ± 38,627[a]	ND	0.72
(E)- 2-Methyl-2-penten-1-ol*	8.826 , 1.808	873	ND	80,314 ± 5,005	ND	1.25
1-(2-Methoxypropoxy)-2-propanol	13.989 , 1.472	1029	ND	ND	91,696 ± 20,337	1.03
1,1'-[(1-Methyl-1,2-ethanediyl)bis(oxy)]bis-2-propanol	26.563 , 1.069	1534	ND	ND	240,386 ± 89,665	1.02
1-[1-Methyl-2-(2-propenyloxy)ethoxy]-2-propanol	26.563 , 0.931	1534	501,399 ± 132,351[a]	238,160 ± 76,644[b]	376,955 ± 110,692[a,b]	0.18
1-[2-(2-Methoxy-1-methylethoxy)-1-methylethoxy]-2-propanol	26.563 , 0.911	1534	410,731 ± 77,826[b]	926,728 ± 106,168[a]	1,327,305 ± 142,222[a]	0.97
1-Ethoxy-2-propanol	20.901 , 1.069	1256	267,648 ± 77,877[a]	53,100 ± 6,268[c]	115,334 ± 38,386[b]	0.56
1-Methoxy-2-propanol	4.080 , 1.201	726	2,199,777 ± 609,617[a]	618,078 ± 43,905[b]	171,020 ± 39,625[c]	1.31
2-Propen-1-ol*	2.665 , 1.294	<500	117,255 ± 34,761	ND	ND	1.18
4-Penten-1-ol	5.746 , 2.013	778	788,030 ± 19,272	ND	ND	1.39
2,6-Dimethyl-7-octen-2-ol*	15.738 , 1.036	1082	119,326 ± 17,912	ND	ND	1.39
Benzyl Alcohol*	14.656 , 4.567	1051	177,433 ± 29,608	ND	ND	1.39
Cyclohexanol*	9.826 , 1.630	903	71,197 ± 20,705	ND	ND	1.37
1-Methyl-cyclopentanol	12.574 , 1.333	985	69,798 ± 25,588	ND	ND	1.16
6-Methyl-5-hepten-2-ol	12.907 , 1.063	995	102,382 ± 28,111	ND	ND	1.01
Ethanol*	2.332 , 0.719	<500	41,423,524 ± 13,025,180[a,b]	67,578,505 ± 20,625,418[a]	24,488,924 ± 2,117,160[b]	0.90
2-(2-Ethoxyethoxy)-ethanol*	13.740 , 2.099	1021	368,845 ± 37,238[b]	410,607 ± 14,870[b]	1,065,663 ± 466,501[a]	1.02
2-Ethoxy-ethanol*	4.830 , 1.670	749	341,250 ± 60,862[a]	284,703 ± 14,940[a,b]	230,049 ± 28,367[b]	0.54
Aldehydes						
(E,E)-2,4-Heptadienal*	13.323 , 1.432	1008	ND	ND	144,319 ± 47,927	1.17
2-Butenal*	3.664 , 0.957	640	6,506,201 ± 1,824,499[a]	4,162,658 ± 579,589[a]	3,719,455 ± 3,928,768[a]	0.93
(E)-2-Methyl-2-butenal	5.329 , 1.016	764	8,091,327 ± 496,909[a]	6,333,043 ± 783,411[b]	3,036,519 ± 1,517,737[c]	1.23
5-Methyl-2-furancarboxaldehyde	12.157 , 2.297	974	ND	ND	2,252,560 ± 1,519,815	1.16
(Z)-2-Heptenal*	11.991 , 1.168	968	85,998 ± 7,370[b]	87,452 ± 12,425[b]	544,307 ± 222,027[a]	1.00
2-Hexenal*	8.577 , 1.096	865	90940 ± 31045[b]	83002 ± 31576[b]	1166732 ± 535842[a]	1.10
(E)-2-Octenal*	15.322 , 1.082	1070	ND	ND	112,330 ± 64,160	1.19
(E)-2-Pentenal*	5.746 , 1.096	777	ND	75,357 ± 610[b]	467,922 ± 142,976[a]	0.64
2-Methyl-2-pentenal	7.827 , 0.964	841	ND	ND	142,959 ± 301	0.94

1,3,4-Trimethyl-3-cyclohexene-1-carboxaldehyde	13.573 , 0.627	1016	ND	116,505 ± 31,559	ND	1.01
3-Furaldehyde*	7.411 , 2.653	829	ND	ND	360,284 ± 166,491	1.02
Acetaldehyde*	2.165 , 1.531	<500	48,289,186 ± 4,040,617[a]	25,367,295 ± 897,056[b]	32,889,951 ± 12,706,387[b]	0.43
Benzaldehyde*	12.157 , 1.921	973	1,192,410 ± 222,535[b]	3,428,161 ± 403,518[b]	9,762,308 ± 3,489,438[a]	1.12
Methyl-benzeneacetaldehyde	15.072 , 0.792	1062	170,999 ± 36,758[a]	74,201 ± 1,761[b]	ND	0.84
Furfural*	7.911 , 2.897	845	2,471,459 ± 963,835[b]	2,733,296 ± 128,520[b]	169,460,381 ± 32,606,122[a]	1.20
Heptanal*	10.076 , 0.851	910	ND	ND	1,712,076 ± 331,134	0.94
Hexanal*	6.911 , 0.858	813	ND	ND	2,309,552 ± 918,639	0.99
2-Ethyl-hexanal	9.076 , 0.686	880	ND	55,232 ± 12,802	ND	1.00
Nonanal*	16.737 , 0.878	1114	ND	ND	365,315 ± 220,516	0.73
Octanal*	13.490 , 0.931	1013	40,798 ± 10,155[b]	ND	565,021 ± 204,249[a]	1.07
Pentanal*	4.330 , 0.772	734	ND	ND	62,540 ± 19,165	1.13
2-Methyl-pentanal*	5.496 , 0.726	769	ND	1,243,331 ± 60,292[a]	763,428 ± 102,252[b]	0.96
Aromatic hydrocarbons						
2,6-Bis(1,1-dimethylethyl)-4-(1-oxopropyl)phenol	27.729 , 0.746	1637	262,088 ± 44,377[a]	194,103 ± 44,235[a]	137,213 ± 7,568[a]	0.86
2,6-Diisopropylnaphthalene	28.811 , 0.832	1756	ND	48,593 ± 12,781[a]	51,452 ± 18,742[a]	1.17
Azulene	19.319 , 1.544	1201	ND	487,283 ± 13,466[a]	519,394 ± 33,317[a]	1.16
Benzene	4.330 , 0.653	734	10,099,523 ± 1,004,380[a]	10,124,767 ± 495,654[a]	8,624,250 ± 632,667[a]	0.99
(1-Methylethyl)-benzene*	10.825 , 0.818	933	212,649 ± 46,269[b]	ND	181,481 ± 62,351[a]	0.87
(1-Methylpropyl)-benzene*	15.155 , 0.799	1064	ND	ND	338,025 ± 9,720	0.94
1,2,3,5-Tetramethyl-benzene*	17.237 , 0.911	1131	ND	133,757 ± 6,064	ND	1.02
1,2,3-Trimethyl-benzene*	13.157 , 0.911	1003	168,497 ± 2,736[b]	607,082 ± 54,956[a]	842,422 ± 317,632[a]	0.20
1,2,4-Trimethyl-benzene*	13.157 , 0.878	1003	137,340 ± 51,828	ND	ND	1.11
1,3-Bis(1,1-dimethylethyl)-benzene*	20.984 , 0.700	1259	358,379 ± 101,544[a]	97,196 ± 21,261[b]	84,106 ± 21,360[b]	0.57
1,3-Dichloro-benzene*	13.573 , 1.168	1016	120,684 ± 37,464[b]	109,077 ± 29,422[b]	464,887 ± 11,097[a]	0.47
1,3-Diethyl-benzene*	14.905 , 0.799	1057	44,079 ± 1,711	ND	ND	1.26
(E)-1-Butenyl-benzene*	16.154 , 0.891	1095	25,317 ± 2,291	ND	ND	0.91
1-Ethyl-2-methyl-benzene	12.491 , 0.832	983	245,452 ± 95,091[a]	174,816 ± 87,609[a]	292,121 ± 91,128[a]	0.87
1-Ethyl-3-methyl-benzene	12.074 , 0.832	970	147,916 ± 60,080[b]	183,794 ± 6,092[a]	221,321 ± 37,053[a]	0.43
1-Ethyl-4-methyl-benzene	12.407 , 0.838	980	ND	ND	492,473 ± 82,252	0.89
1-Methyl-2-(1-methylethyl)-benzene*	14.322 , 0.746	1039	1,539,400 ± 527,933[a]	1,926,339 ± 262,421[a]	1,475,444 ± 145,824[a]	0.75

1-Methyl-3-(1-methylethyl)-benzene*	14.073 , 0.785	1031	78,186 ± 37,794	ND	ND	1.06
1-Methyl-4-(1-methylethenyl)-benzene*	16.321 , 1.049	1100	47,689 ± 355[b]	53,089 ± 2,008[b]	71,335 ± 13,886[a]	0.61
1-Methyl-4-propyl-benzene	13.653 , 0.785	1018	248,070 ± 26,816	ND	ND	1.16
2-Ethyl-1,4-dimethyl-benzene*	15.821 , 0.832	1085	395,174 ± 66,367[a]	348,108 ± 196,060[a]	ND	0.64
2-Propenyl-benzene*	11.575 , 0.931	955	ND	191,494 ± 48,747	ND	1.22
Chloro-benzene*	8.327 , 1.063	857	315,617 ± 80,989[a]	295,427 ± 112,074[a]	ND	1.08
Cyclopropyl-benzene	11.575 , 0.931	955	388,378 ± 71,225	ND	ND	1.01
Methoxy-benzene	10.575 , 1.261	925	ND	ND	40,394 ± 5,145	0.94
Propyl-benzene*	11.908 , 0.799	965	265,503 ± 93,278[a]	148,382 ± 93,659[a]	126,663 ± 64,280[a]	0.65
Benzeneacetaldehyde*	14.822 , 1.934	1055	351,375 ± 45,872[b]	1,495,378 ± 68,235[a]	1,901,142 ± 459,923[a]	1.21
Ethylbenzene*	8.743 , 0.851	870	319,987 ± 99,757[b]	249,758 ± 36,117[b]	3,557,512 ± 191,075[a]	1.15
Naphthalene	19.235 , 1.577	1198	454,687 ± 42,431	ND	ND	1.02
[2R-(2α,4α,8α)]-1,2,3,4,4’,5,6,8-octahydro-4,8-dimethyl-2-(1-methylethenyl)-naphthalene	25.148 , 0.614	1437	272,800 ± 98,768	ND	ND	1.16
trans-Decahydro-naphthalene	15.405 , 0.601	1072	97,552 ± 43,066	ND	ND	0.96
Phenylethyne*	9.2430 , 1.558	885	59,535 ± 14,495[b]	72,437 ± 13,672[b]	120,930 ± 3,790[a]	0.79
p-Xylene*	8.993 , 0.858	877	1,882,477 ± 643,346[a]	775,055 ± 178,417[a]	4,645,281 ± 2,103,045[a]	1.01
Styrene*	9.743 , 1.115	900	3,570,731 ± 456,076[b]	14,199,922 ± 2,042,810[a]	10,019,356 ± 953,491[a,b]	0.95
Toluene*	5.995 , 0.792	785	99,574,268 ± 13,314,333[a]	92,559,263 ± 15,905,679[a]	101,808,335 ± 15,198,835[a]	0.85
Esters						
1,2-Benzenedicarboxylic acid, bis(2-methylpropyl) ester*	29.811 , 1.261	1880	1,006,575 ± 148,520[a]	1,165,587 ± 123,744[a]	1,215,658 ± 188,374[a]	0.88
1-Methoxy-2-propyl acetate	8.993 , 0.997	877	ND	ND	351,997 ± 87,836	1.18
2,2,4-Trimethyl-1,3-pentanediol diisobutyrate	23.732 , 1.412	1366	281,405 ± 19,326[b]	ND	416,503 ± 39,274[a]	0.85
2-Propenoic acid, 2-methyl-, methyl ester	4.663 , 0.779	744	113,600 ± 20,897	ND	ND	1.39
2-Propyn-1-ol, propionate	2.664 , 1.287	<500	ND	76,532 ± 24,470	ND	1.15
3-Methylheptyl acetate*	17.986 , 0.766	1156	292,295 ± 26,715	ND	ND	0.97
Acetic acid, butyl ester	7.328 , 0.785	826	203,527 ± 63,043[b]	ND	17,103,261 ± 8,752,483[a]	0.64
Alpha-amino-gamma-butyrolactone	7.078 , 0.403	818	ND	700,982 ± 26,310	ND	1.03
Benzoic acid, methyl ester*	16.488 , 1.538	1106	198,198 ± 74,929[b]	272,183 ± 73,960[a,b]	353,331 ± 114,353[a]	0.83
Butanedioic acid, dimethyl ester	14.406 , 1.729	1042	ND	58,623 ± 8,562[a]	75,326 ± 23,341[a]	1.02
Butyrolactone*	10.492 , 3.544	924	12,343,269 ± 889,045[a]	10,682,166 ± 417,100[a,b]	7,603,298 ± 1,685,471[b]	1.23

Cyclopentaneacetic acid, 3-oxo-2-pentyl-, methyl ester*	28.062 , 0.983	1674	93,319 ± 2,850[b]	120,397 ± 6,515[a,b]	157,593 ± 65,336[a]	0.94
Dibutyl phthalate*	30.560 , 1.492	1981	ND	371,691 ± 62,759[a]	344,206 ± 85,023[a]	1.22
Diethyl Phthalate*	27.396 , 1.261	1602	64,514 ± 3,388[a]	81,931 ± 3,122[a]	89,247 ± 39,662[a]	0.93
Ethyl Acetate	10.492 , 0.812	923	55,246,893 ± 6,854,404[a]	56294576 ± 1038507[a]	47,490,603 ± 7,915,048[a]	0.50
Ethyl acetoacetate ethylene acetal*	18.236 , 1.379	1165	38,626 ± 1,995	ND	ND	1.26
Oxalic acid, allyl octadecyl ester	25.564 , 0.574	1464	420,749 ± 145,096	ND	ND	0.98
Pentafluoropropionic acid, tetradecyl ester	14.489, 0.561	1044	ND	ND	597,965 ± 335,495	0.78
Pentanoic acid, 10-undecenyl ester	12.491 , 0.561	983	ND	ND	789,930 ± 201,559	0.95
Propanoic acid, 2-hydroxy-, ethyl ester	7.411 , 1.195	829	ND	168,401 ± 4,082	ND	0.90
Propanoic acid, 2-methyl-, 2,2-dimethyl-1-(2-hydroxy-1-methylethyl)propyl ester	23.815 , 1.445	1370	1,264,715 ± 139,689[a]	874,907 ± 431,769[a]	ND	1.18
Propanoic acid, 2-methyl-, 3-hydroxy-2,4,4-trimethylpentyl ester	24.232 , 1.076	1387	865,060 ± 263,514[a]	678,147 ± 227,979[a,b]	409,270 ± 291,617[b]	0.81
Propanoic acid, 2-oxo-, methyl ester	4.996 , 1.709	755	ND	ND	70,567 ± 2,370	0.89
Triacetin*	23.482 , 1.960	1356	1,782,542 ± 188,162[a]	2,162,732 ± 111,706[a]	1,965,252, ± 95,657[a]	0.90
Ethers						
cis-2,3-Epoxyoctane	11.824 , 0.739	963	271,540 ± 126,645[b]	1,195,026 ± 123,393[a]	ND	1.11
Dimethyl ether*	2.2483 , 0.726	<500	1,059,524 ± 246,819[b]	1,751,937 ± 698,540[b]	4,557,365 ± 2,118,353[a]	0.34
2-Methyl-2-propyl-oxirane	5.413 , 0.653	767	ND	2,058,104 ± 218,233[a]	269,705 ± 5,166[b]	1.41
Tetramethyl-oxirane	5.829 , 1.096	780	237,420 ± 49,086	ND	ND	1.01
trans-3,4-Epoxyoctane	12.324 , 0.733	978	55,845 ± 11,302[b]	763,649 ± 307,030[a]	414,802 ± 143,586[a]	0.45
Hydrocarbons						
1,2-15,16-Diepoxyhexadecane	27.396 , 0.554	1601	ND	159,104 ± 42,869	ND	1.13
1,2-Dipentylcyclopropene	18.902 , 0.607	1186	32,093 ± 6,112	ND	ND	0.99
1,3,5,7-Cyclooctatetraene	9.826 , 1.049	903	2,509,440 ± 1,476,921[a]	424,812 ± 94,805[b]	ND	0.55
1,3,5-Cycloheptatriene	6.995 , 0.964	816	ND	14,268,473 ± 1,488,125	ND	0.65
1-Methyl-4-(1-methylethyl)-1,4-cyclohexadiene*	13.989 , 0.726	1028	82,054 ± 3,918[a]	90,331 ± 6,651[a]	ND	0.73
3,3,4,4-Tetrafluoro-1,5-hexadiene	32.558 , 0.449	2245	456,071 ± 136,843[a]	440,360 ± 124,538[a]	ND	1.13
3,5,5-Trimethyl-1-hexene*	5.912 , 0.469	782	6,160,274 ± 1,237,716[a]	3,765,696 ± 797,472[b]	7,175,432 ± 838,686[a]	0.68
3,5-Dimethyl-1-hexene	6.162 , 0.442	790	ND	49,930 ± 3,298	ND	1.09
4-Methyl-1-hexene	3.830 , 0.442	718	723,076 ± 338,336	ND	ND	0.80
1-Iodo-2-methylundecane	26.146 , 0.535	1500	485,675 ± 209,515[a]	400,375 ± 197,177[a]	834,411 ± 225,563[a]	0.71

4,6,8-Trimethyl-1-nonene	23.565 , 0.561	1359	ND	112,243 ± 29,372	ND	0.98
3,7-Dimethyl-1-octene	14.739 , 0.541	1051	ND	223,262 ± 64,861	ND	0.75
1-Pentene*	2.748 , 0.422	501	11,532,252 ± 495,174[a]	ND	156,513 ± 363[b]	0.81
2,4,4-Trimethyl-1-pentene	4.663 , 0.462	744	54,016 ± 5,722[a,b]	49,316 ± 2,938[b]	62,877 ± 1,047[a]	0.61
4-Methyl-1-undecene*	16.321 , 0.521	1100	79,147 ± 9,191[a]	ND	158,320 ± 31,376[a]	0.77
2,4,6,8-Tetramethyl-1-undecene	14.489 , 0.541	1044	313627 ± 112305[a]	219,231 ± 111,301[a]	229,365 ± 63,170[a]	0.31
2,4,6-Trimethyl-1-nonene	8.077, 1.406	849	184147 ± 28856[a]	267429 ± 43913[a]	ND	1.24
(Z,Z)-2,4-Hexadiene	3.581 , 0.528	710	969,368 ± 226,433	ND	ND	1.33
2-Cyclopentene-1,4-dione	9.493 , 3.584	894	ND	ND	851,967 ± 293,620	1.17
2-Methyl-1,5-heptadiene	9.326 , 1.577	888	ND	ND	5,295,155 ± 1,096,105	1.18
4-Methyl-3-heptene	5.579 , 0.462	772	ND	ND	707,303 ± 430,480	0.95
(Z)-3-Octene	7.078 , 0.495	818	3,305,857 ± 176,709	ND	ND	0.78
4-Methyl-1,4-heptadiene	17.236 , 0.937	1131	71,016 ± 11,311[a]	36,642 ± 4,004[b]	ND	0.82
(E)-4-Octene*	6.578 , 0.488	803	152,530 ± 33,125[a]	217,370 ± 154,254[a]	ND	0.62
(Z)-5-Tridecene	15.905 , 0.554	1087	ND	ND	261,675 ± 50,302	1.02
9-Octadecyne	18.403, 0.561	1170	ND	51,996 ± 23,976	ND	0.95
2-Nitro-butane	12.657 , 2.013	988	59,509 ± 10,578[b]	989,281 ± 267,413[b]	36,998,646 ± 13,089,861[a]	1.24
cis-1,3-Dimethyl-2-methylene-cyclohexane	5.246 , 0.469	762	ND	137,917 ± 1,949	ND	1.03
1-Ethyl-2-propyl-cyclohexane	14.905 , 0.548	1057	ND	141,552 ± 6,936	ND	1.03
(S)-1-Methyl-4-(1-methylethenyl)-cyclohexene	14.322 , 0.700	1039	1,331,006 ± 532,580[a]	1,458,834 ± 279,764[a]	1,715,713 ± 624,007[a]	0.62
1-Methyl-4-(1-methylethylidene)-cyclohexene	13.906 , 0.680	1026	59,172 ± 14,886[a]	68,889 ± 1,858[a]	ND	0.93
1-Methyl-3-(2-methylpropyl)-cyclopentane	12.907, 0.528	995	ND	113,624 ± 28,851	ND	1.14
Methyl-cyclopentane	3.331 , 0.502	703	18,392,760 ± 244,343[a]	17,740,139 ± 462,822[a]	17,835,420 ± 59,524[a]	0.68
Ethyl-cyclopropane	2.748 , 0.422	501	ND	3,309,830 ± 160,408	ND	1.04
Decane*	13.406 , 0.508	1021	1,204,851 ± 442,636[a]	415,718 ± 126,781[b]	318,437 ± 147,578[b]	0.69
2,3,5,8-Tetramethyl-decane	26.313 , 0.541	1514	112,414 ± 27,748[b]	171,795 ± 43,947[b]	679,482 ± 224,503[a]	0.73
5-Methyl-decane*	13.989 , 0.521	1028	205,849 ± 75,602[a]	189,828 ± 40,958[a]	ND	0.84
Dodecane*	18.236 , 0.541	1121	397,029 ± 111,602	ND	ND	0.94
2,6,10-Trimethyl-dodecane	17.403 , 0.561	1136	ND	76,707 ± 34,132	ND	1.16
2,6,11-Trimethyl-dodecane	18.819 , 0.568	1184	176,155 ± 81,345[b]	108,286 ± 9,859[b]	326,525 ± 9,169[a]	0.66
Pentadecane*	22.399 , 0.574	1488	ND	441,191 ± 190,020[a]	330,649 ± 71,282[a]	1.12

2,2,4,6,6-Pentamethyl-heptane	12.990 , 0.495	998	103,855 ± 8,841[b]	163,915 ± 75,028[b]	593,410 ± 258,764[a]	0.84
2,4-Dimethyl-heptane*	7.744 , 0.462	839	ND	3,770,336 ± 132,716	ND	0.87
3-Ethyl-heptane	12.074 , 0.508	970	ND	635,101 ± 194,390	ND	0.95
3-Methylene-heptane	7.078 , 0.561	818	ND	2,938,287 ± 872,460	ND	0.84
Hexadecane*	21.900 , 0.535	1600	360,036 ± 40,675[a]	237,373 ± 126,281[a]	322,217 ± 37,783[a]	0.82
Hexane*	3.081 , 0.495	605	217,219,667 ± 21,244,985[a]	218,411,828 ± 34,932,339[a]	219,892,423 ± 56,504,217[a]	0.67
2,4-Dimethyl-hexane	7.494 , 0.475	831	4,801,953 ± 959,184[a]	7,213,514 ± 1,530,301[a]	ND	0.78
Nitro-methane	2.748 , 1.478	685	115,379 ± 8,610[a]	89,860 ± 3,704[a,b]	66,836 ± 2,197[b]	0.60
Trimethoxy-methane	2.998 , 0.884	693	62,681 ± 12,101[a]	73,701 ± 34,956[a]	ND	0.90
Tetradecane*	23.649 , 0.587	1382	212,661 ± 78,339	ND	ND	1.08
Nonane*	9.992 , 0.495	908	1,956,428 ± 305,345[a]	2,307,334 ± 555,086[a]	2,172,996 ± 722,228[a]	0.65
2,6-Dimethyl-nonane*	14.073 , 0.528	1031	ND	552,641 ± 165,769	ND	1.18
2-Methyl-nonane*	12.157 , 0.515	973	483,434 ± 129,077	ND	ND	0.98
2,3,6,7-Tetramethyl-octane	16.987 , 0.528	1122	60,831 ± 7,996[c]	613,503 ± 257,357[a]	164,508 ± 69,367[b]	0.67
2,7-Dimethyl-octane*	10.492 , 0.700	923	928,245 ± 388,138[a]	1,609,453 ± 303,526[a]	1,431,521 ± 297,042[a]	1.00
3,5-Dimethyl-octane	13.573 , 0.508	1016	289,164 ± 138,612[a]	257,499 ± 24,973[a]	ND	0.80
4-Methyl-octane*	8.827 , 0.488	872	3,690,807 ± 552,610[a]	3,890,642 ± 1,730,785[a]	3,365,886 ± 259,747[a]	0.68
2,2-Dimethyl-pent-4-yn-3-one*	4.413 , 1.353	736	ND	48,479 ± 1,475	ND	1.43
1-Nitro-pentane	11.491 , 1.346	953	141,710 ± 17,770[a]	126,220 ± 3,960[a]	ND	1.12
3-Methyl-pentane	2.831 , 0.449	687	11,505,205 ± 1,254,620[a]	11,307,042 ± 282,382[a]	9,794,382 ± 2,323,123[a]	0.67
1,2-Dimethoxy-propane	13.989 , 1.478	1029	66,350 ± 13,633[a]	59,516 ± 3,061[a]	ND	0.86
Propene	3.081 , 0.904	695	75,323 ± 20,320	ND	ND	0.91
Spiro[2.4]hepta-4,6-diene	6.245 , 0.858	793	69,000 ± 19,965[a]	53,399 ± 7,484[a]	ND	0.84
Tetradecane	18.735 , 0.548	1181	ND	163,901 ± 46,949[b]	495,226 ± 121,723[a]	0.76
Undecane*	16.570 , 0.601	1109	505,769 ± 37,964[b]	792,251 ± 332,291[a,b]	851,911 ± 190,155[a]	0.78
2,6-Dimethyl-undecane*	19.984 , 0.554	1224	ND	81,939 ± 21,244[a]	89,053 ± 26,315[a]	0.80
2-Methyl-undecane*	18.486 , 0.554	1172	76,388 ± 38,322[a]	119,462 ± 62,515[a]	ND	0.60
4,7-Dimethyl-undecane	14.572 , 0.521	1046	48,952 ± 20,058	ND	ND	0.87
4-Methyl-undecane*	16.987 , 0.548	1122	ND	150,645 ± 101,313	ND	0.85
Isothiocyanates						
4-Isothiocyanato-1-butene	12.740 , 1.439	991	2,424,537 ± 173,379[a]	404,829 ± 99,138[b]	89,076 ± 16,791[b]	1.36

Allyl Isothiocyanate	9.493 , 1.518	893	103,194 ± 10,966	ND	ND	0.76
Ketones						
1-Hydroxy-2-butanone	6.079 , 2.772	788	ND	ND	429,851 ± 271,131	0.80
1-Penten-3-one	4.163 , 0.845	729	ND	805,599 ± 919	ND	0.82
2,3-Butanedione	4.163 , 0.733	728	171,348 ± 56,811	ND	ND	1.33
2,3-Pentanedione	12.074 , 1.914	971	ND	ND	1,699,776 ± 33,232	1.13
2,5-Hexanedione*	10.992 , 2.092	938	399,986 ± 40,758[b]	667,950 ± 31,080[a]	401,351 ± 103,961[b]	1.24
3-Hydroxy-2-butanone*	4.663 , 2.185	745	687,911 ± 287,842[b]	ND	5,756,483 ± 1,565,925[a]	1.23
3-Methyl-2-butanone	4.163 , 0.733	728	ND	199,922 ± 7,133	ND	0.53
2-Cyclopenten-1-one*	7.911 , 1.855	844	59,921 ± 8,647[b]	201,019 ± 7,159[b]	3,170,894 ± 440,713[a]	1.20
2-Methyl-2-cyclopenten-1-one	10.242 , 1.465	916	ND	ND	252,391 ± 44,435	1.20
3-Methyl-2-cyclopenten-1-one	4.663 , 0.686	744	ND	ND	953,865 ± 139,476	0.89
3-Methyl-2-heptanone	11.491 , 0.799	953	ND	59,991 ± 25,099	ND	0.99
4-Methyl-2-heptanone*	11.325 , 0.825	948	139,480 ± 29,473[a]	ND	191,432 ± 73,883[a]	1.04
2-Hexanone*	6.578 , 0.865	803	52,680,335 ± 7,421,129[b]	91,737,416 ± 3,632,239[a]	15,217,463 ± 8,827,113[c]	1.21
3-Methyl-4-methylene-2-hexanone	13.157 , 0.970	1003	ND	198,757 ± 86,952	ND	0.64
2-Hydroxy-3-pentanone	7.161 , 2.026	821	ND	ND	568,009 ± 58,217	1.19
2-Octanone*	13.073 , 0.904	1000	861,377 ± 169,948[a]	820,146 ± 34,520[a]	867,026 ± 33,603[a]	0.68
3-Methylene-2-pentanone	6.828 , 0.964	811	ND	4,221,734 ± 690,884	ND	1.19
4-Hydroxy-4-methyl-2-pentanone	8.410 , 1.723	860	478,248 ± 68,560[b]	416,377 ± 14,764[b]	1,226,126 ± 247,993[a]	1.10
1-Hydroxy-2-propanone*	3.914 , 2.620	722	ND	1,106,767 ± 35,883[b]	10,333,493 ± 243632[a]	1.29
1-Methoxy-2-propanone	4.247 , 1.109	731	1,734,245 ± 466,156[a]	1,449,729 ± 371,929[a]	ND	1.03
(E,E)-3,5-Octadien-2-one	9.493 , 0.785	893	120,083 ± 7,403[b]	168,728 ± 5,764[a,b]	221,601 ± 4,828[a]	0.98
3-Heptanone*	9.576 , 0.858	895	143,939 ± 35,703[b]	185,863 ± 12,428[a,b]	214,752 ± 20,884[a]	0.89
4-Methyl-3-hepten-2-one	13.822, 0.950	1023	ND	702,103 ± 73,727	ND	1.02
3-Hexanone*	6.412 , 0.818	798	972,545 ± 113,221[a]	140,380 ± 73,476[b]	ND	0.57
3,4-Dimethyl-3-hexen-2-one	13.822 , 0.944	1023	69,033 ± 16,449	ND	ND	0.92
(E)-3-Octen-2-one*	14.656 , 1.129	1049	521,099 ± 36,685[b]	579,407 ± 67,123[b]	1,000,529 ± 331,961[a]	0.96
(E)-3-Penten-2-one	5.329 , 1.082	765	1,721,080 ± 150,558[a]	1,896,384 ± 382,027[a]	2,241,306 ± 631,216[a]	0.85
3-Methyl-3-penten-2-one	8.077 , 1.043	849	125,521 ± 46,311[a]	154,255 ± 21,853[b]	ND	0.52
4-Methyl-3-penten-2-one	6.828 , 0.964	811	8,172,591 ± 738,090	ND	ND	1.20

6-Methyl-5-hepten-2-one*	12.907 , 1.010	995	256,019 ± 42,361[b]	175,990 ± 28,316[b]	2,793,117 ± 846,163[a]	1.03
5-Hexen-2-one	14.822 , 1.188	1054	ND	ND	487,016 ± 56,074	0.96
Acetone*	2.415 , 0.554	<500	66,738,480 ± 12,705,734[a]	76,574,287 ± 1,401,668[a]	68,464,003 ± 1,632,666[a]	0.81
Acetophenone*	15.572 , 1.868	1078	343,758 ± 13,847[b]	666,714 ± 127,712[a]	614,914 ± 21,081[a]	1.06
Cyclohexanone*	9.909 , 1.214	905	ND	267,485 ± 51,196	ND	1.17
trans-2-Methyl-5-(1-methylethenyl)-cyclohexanone*	19.818 , 1.109	1218	46,451 ± 2,724[b]	ND	188,666 ± 59,075[a]	0.89
1-(1H-pyrrol-2-yl)-ethanone	15.738 , 0.363	1082	ND	ND	425,530 ± 186,350	1.15
1-(2-Furanyl)-ethanone	10.409 , 2.270	921	ND	ND	3,472,605 ± 93,247	1.17
Nitrogen-containing compounds						
N-[4-Bromo-*n*-butyl]-2-piperidinone	25.730 , 0.568	1474	542,352 ± 165,112[a]	600,676 ± 136,109[a]	693,673 ± 50,392[a]	0.71
1-Methyl-2-pyrrolidinone*	15.238 , 2.119	1068	ND	ND	272,635 ± 21,443	1.14
2-Methyl-3-butyn-2-amine	11.241 , 1.591	946	ND	ND	134,261 ± 3,125	0.95
3-Nitro-2-methyl propene	9.409 , 1.518	890	ND	ND	558,028 ± 116,931	1.26
Cyclobutylamine	2.498 , 0.455	<500	ND	194,131 ± 4,398	ND	0.92
Dimethylamine	4.579 , 1.155	741	380,423 ± 57,678[a]	385,158 ± 39,811[a]	ND	0.90
Endo-3-acetamidocamphor	2.747 , 1.703	<500	38,243 ± 7,339[a]	40,528 ± 2,728[a]	ND	0.73
N,N-Dimethyl-formamide	7.244, 1.802	824	ND	ND	400,307 ± 49,563	1.21
Hydrogen azide	6.162 , 2.614	791	236,433 ± 93,495[b]	795,679 ± 328,526[a]	795,098 ± 205,356[a]	1.04
O-Methyl-hydroxylamine	4.163 , 1.188	729	98,165 ± 43,335[a]	58,922 ± 2,851[a]	ND	1.07
Bis(1,1-dimethylethyl)nitroxide	10.242, 0.647	916	103,754 ± ,9479	ND	ND	0.91
Pyrazine*	5.246 , 1.452	762	452,448 ± 75,536[b]	299,872 ± 60,672[b]	8,286,886 ± 1,660,434[a]	1.19
2,5-Dimethyl-pyrazine*	10.492 , 1.221	923	1,222,095 ± 568,332[b]	1,183,469 ± 309,167[b]	21,617,858 ± 682,009[a]	1.16
2-Ethyl-3-methyl-pyrazine*	13.573 , 1.115	1016	291,156 ± 77,106[b]	ND	1,735,020 ± 139,196[a]	1.13
2-Ethyl-5-methyl-pyrazine	13.739 , 1.063	1021	ND	137,874 ± 1,395	ND	1.03
2-Ethyl-6-methyl-pyrazine*	13.407 , 1.089	1011	ND	ND	250,708 ± 12,043	1.06
3-Ethyl-2,5-dimethyl-pyrazine*	15.905 , 1.023	1088	719,596 ± 363,314[a,b]	385,884 ± 94,190[b]	1,115,778 ± 518,734[a]	0.74
Methyl-pyrazine*	7.660 , 1.353	836	1,446,052 ± 214,740[b]	1,009,692 ± 31,639[b]	49,667,411 ± 637,672[a]	1.19
Trimethyl-pyrazine*	13.739 , 1.102	1021	ND	ND	85,184 ± 19,818	0.92
Pyridine*	5.745 , 1.228	777	345,906,289 ± 115,290,603[b]	480,089,342 ± 50,193,973[a]	377,909,769 ± 63,388,776[a,b]	0.95
Nitriles						

2,4-Hexadienenitrile	11.075 , 1.993	941	ND	ND	167,496 ± 74,892	1.08
2,4-Pentadienenitrile	6.411 , 2.006	798	3,173,720 ± 515,538[b]	2,360,293 ± 201,374[b]	23,070,929 ± 6,013,054[a]	1.18
2-Butenenitrile*	4.080 , 1.373	726	ND	ND	1,552,890 ± 313,787	1.19
2-Furancarbonitrile	6.911 , 2.442	814	ND	ND	593,251 ± 6,326	1.18
2-Pentenenitrile	5.828 , 1.802	780	277,978 ± 74,251[a]	382,734 ± 180,124[a]	ND	0.86
3-Butenenitrile*	3.913 , 1.511	721	191,780 ± 48,551[c]	1,417,915 ± 269,028[a]	757,808 ± 216,530[b]	0.90
3-Pentenenitrile	2.581 , 0.851	<500	53,172,285 ± 5,516,095[b]	5,5056,554 ± 2,325,393[c]	92,380,768 ± 13,583,216[a]	1.17
2-Methyl-5-hexenenitrile	8.743 , 1.650	870	9,617,079 ± 2,200,752[b]	6,530,657 ± 2,3964[b]	79,267,131 ± 131,602[a]	1.20
Acetonitrile*	2.415 , 0.891	<500	85,924,582 ± 11,852,726[a]	77,494,809 ± 17,968,539[a,b]	59,224,353 ± 11,100,114[b]	0.91
Benzenepropanenitrile*	20.734 , 2.878	1252	179,696 ± 83,125[a]	ND	205,103 ± 14,648[a]	1.12
Benzonitrile*	12.907 , 2.284	996	64,799 ± 7,887[b]	55,855 ± 9,430[b]	127,131 ± 46,828[a]	0.95
Benzyl nitrile*	17.737 , 3.208	1149	ND	ND	145,940 ± 560	1.16
Butanenitrile	6.911 , 1.551	813	ND	ND	96,641 ± 673	0.94
Ethanedinitrile	12.074 , 0.792	970	ND	69,596 ± 11,380	ND	0.96
Hexanenitrile	11.741 , 1.214	960	250,507 ± 89,455[b]	ND	845,763 ± 187,586[a]	0.96
5-Methyl-hexanenitrile	11.574 , 1.214	955	226,577 ± 109,641[b]	159,173 ± 76,366[b]	717,470 ± 91,944[a]	1.11
Methallyl cyanide	5.745 , 1.782	778	ND	ND	758,888 ± 217,437	1.09
Methyl isocyanide	2.414 , 0.878	<500	11,108,821 ± 4,097,400[a]	13,875,092 ± 1,188,730[a]	ND	0.85
4-Methyl-pentanenitrile	8.160 , 1.208	852	ND	160,890 ± 5,981	ND	1.26
O-heterocyclic compounds						
1,3,6-Trioxocane	6.828 , 0.772	811	1,303,652 ± 556,574[a]	753,832 ± 201,378[a]	1,436,974 ± 2,314,010[a]	0.77
4,7-Dihydro-1,3-dioxepin	14.572 , 3.788	1048	ND	ND	5,616,646 ± 2,507,763	1.01
1,3-Dioxolan-2-one	7.994 , 0.719	846	ND	ND	179,714 ± 52,504	0.92
2-Ethyl-1,3-dioxolane	5.495 , 0.812	770	848,161 ± 194,832[a]	819,740 ± 41,071[a]	ND	1.26
1,4-Dioxane	4.663 , 0.937	744	331,137,152 ± 17,657,429[a]	311,368,574 ± 11,971,562[a]	258,495,109 ± 9,700,561[b]	1.24
2,3-Dihydro-1,4-dioxin	4.080 , 0.990	726	730,296 ± 93,019[a]	344,650 ± 67,814[b]	250,501 ± 9,003[b]	1.28
5-Ethyldihydro-2(3H)-furanone*	15.155 , 2.534	1065	342,833 ± 30,541[a]	198,445 ± 6,631[b]	238,042 ± 55,697[a,b]	1.20
5-Methyl-2(3H)-furanone*	8.993 , 2.092	878	ND	ND	159,486 ± 72,698	1.22
Dihydro-5-methyl-2(3H)-furanone	4.413 , 1.221	736	171,705 ± 13,660[a]	129,396 ± 4,347[a]	ND	1.29
2(5H)-Furanone	6.245 , 2.845	794	ND	150,322 ± 39,414[b]	12,045,887 ± 6,767,917[a]	1.10
5-Methyl-2(5H)-furanone*	11.325 , 3.973	949	ND	ND	696,971 ± 113,872	1.21

Dihydro-3-methyl-2,5-furandione	14.572 , 3.762	1048	ND	ND	2,853,532 ± 1,125,442	0.89
Dihydro-2-methyl-3(2H)-furanone	7.078, 1.406	819	ND	ND	235,269 ± 23,286	1.22
3,4-Dimethyldihydrofuran-2,5-dione	3.914 , 1.294	678	ND	ND	85,331 ± 6,039	1.06
4-Methyl-5H-furan-2-one	14.822 , 4.772	1056	ND	ND	383,731 ± 19,764	1.15
2-Pentyl-furan*	13.073 , 0.785	1000	376,549 ± 46,654	ND	ND	0.91
Sulfur-containing compounds						
2-Acetyl-5-methylthiophene	20.984 , 0.904	1259	ND	54,209 ± 9,682	ND	1.24
3-Thietanol	5.456 , 1.663	773	ND	43,722 ± 1,436	ND	1.09
4-Methylthiazole*	7.494 , 1.439	831	ND	ND	654,509 ± 253,593	1.12
Carbon disulfide	5.829 , 1.637	780	ND	5,203,471 ± 568,300[a]	7,719,375 ± 2,222,135[a]	1.10
Carbonyl sulfide	2.581 , 0.488	<500	3,204,742 ± 147,985[a]	661,184 ± 66,575[b]	2,448,899 ± 763,295[a]	0.82
Dimethyl sulfide	1.998 , 4.382	<500	124,840,954 ± 24,408,861	ND	ND	1.10
Dimethyl sulfone*	11.074 , 4.231	942	908,682 ± 403,853[b]	1,049,926 ± 405,404[b]	2,050,925 ± 133,328[a]	0.94
Dimethyl Sulfoxide*	8.660 , 3.683	868	2,651,042 ± 328,936[b]	2,248,823 ± 79,557[b]	4,293,094 ± 556,890[a]	0.99
Bis[1-(methylthio)ethyl]disulfide	3.081 , 0.904	695	ND	46,304 ± 531	ND	0.70
2-(Methylthio)-ethanol*	8.160 , 3.755	853	238,141 ± 23,560	ND	ND	1.40
Methanesulfonic anhydride	2.748 , 1.848	500	1,033,581 ± 129,631[a]	ND	215,134 ± 129,679[b]	0.66
Methanethiol*	2.498 , 1.756	678	235,522 ± 180,277[a]	402,296 ± 83,644[a]	301,083 ± 133,944[a]	0.29
Sulfur dioxide	3.248 , 1.709	585	ND	701,715 ± 26,818[a]	190,808 ± 21,949[b]	1.44
Thiazole*	5.413 , 1.683	767	320,390 ± 29,298[b]	417,922 ± 23,167[b]	2,322,206 ± 256,735[a]	1.19
Thiocyanic acid, methyl ester	4.580 , 2.086	742	470,204 ± 60,256[b]	550,116 ± 23,836[b]	3,589,346 ± 187,319[a]	1.21
Terpenes						
(+)-4-Carene*	13.239 , 0.693	1005	ND	ND	46,737 ± 11,520	1.02
[1S-(1α,3α,4α,8α)]- Decahydro-4,8,8-trimethyl-9-methylene-1,4-methanoazulene	25.148 , 0.601	1437	ND	441,827 ± 123,958	ND	0.94
Octahydro-1,4,9,9-tetramethyl-1H-3α,7-methanoazulene	24.981 , 0.607	1427	31,836 ± 1,414[b]	174,774 ± 95,593[a]	ND	0.58
3-Carene*	13.656 , 0.653	1018	ND	358,138 ± 11,193	ND	1.09
α-Phellandrene*	15.322 , 0.733	1070	ND	160,579 ± 5,020	ND	1.09
α-Pinene*	12.657 , 0.640	988	271,918 ± 152,902[b]	548,358 ± 19,663[a]	155,612 ± 24,317[b]	0.91
β-Pinene*	11.158 , 0.554	943	84,527 ± 25,341	ND	ND	0.90
2-Methyl-5-(1-methylethyl)-bicyclo[3.1.0]hex-2-ene	12.491 , 0.673	983	ND	147,749 ± 54,526	ND	1.08

4-Methyl-1-(1-methylethyl)-bicyclo[3.1.0]hexane, didehydro deriv.	12.491 , 0.667	983	170,306 ± 16,911	ND	ND	1.40
2,6,6-Trimethyl-bicyclo[3.1.1]hept-2-ene	11.158 , 0.568	943	187,954 ± 27,590[a]	112,264 ± 4,254[b]	142,950 ± 12,734[a,b]	0.93
(1α,2α,5α)-2,6,6-trimethyl-bicyclo[3.1.1]heptan-3-one	13.739 , 0.647	1021	51,576 ± 4,468	52,245 ± 16,328	ND	0.54
cis-Limonene oxide*	17.6532 , 0.891	1145	ND	51,372 ± 13,286[a]	75,277 ± 15,162[a]	1.16

Different letters in the same row indicate a significant difference ($p < 0.05$) (LSD Fisher, ANOVA).

([1]) RT – retention time; ([2]) LRI – linear retention index. Compounds marked with * had retention indices within 3% of values reported in NIST library.
